# Neuroactive network tissue based on dual-factor neuroregenerative bioactive coating scaffolds and neural stem cells for spinal cord injury repair

**DOI:** 10.1016/j.mtbio.2025.102172

**Published:** 2025-08-05

**Authors:** Tianyi Liu, Wenhao Zhu, Zheng Wan, Cong Fu, Xiaoyu Zhang, Wenzhong Li, Wenchen Li, Zhenxu Wu, Min Guo, Mengtuan Long, Feiyang Yang, Hongyu Chen, Xingcheng Yi, Honglei Wang, Peibiao Zhang, Haifeng Wang

**Affiliations:** a Department of Neurosurgery, The First Hospital of Jilin University, Changchun, 130021, China; b Department of Neurosurgery, The Second Hospital of Jilin University, Changchun, 130041, China; c Department of Neurosurgery, The Second Affiliated Hospital of Zhejiang University School of Medicine, Zhejiang University, Hangzhou, 310016, China; d Key Laboratory of Polymer Ecomaterials, Changchun Institute of Applied Chemistry, Chinese Academy of Sciences, 5625 Renmin Street, Changchun, 130022, China; e Laboratory of Cancer Precision Medicine, First Hospital of Jilin University, Changchun, 130061, China; f National-Local Joint Engineering Laboratory of Animal Models for Human Diseases, Changchun, 130062, China; g Department of Neurosurgery, Shanxi Provincial People's Hospital, The Fifth Hospital of Shanxi Medical University, Taiyuan, 030001, China; h College of Computer Science and Technology, Jilin University, Changchun, 130012, China; i School of Software, Tsinghua University, Beijing, 10084, China; j School of Computer Science, Hangzhou Dianzi University, 310018, China

**Keywords:** Immobilized recombinant growth factors, Spinal cord injury, Neuroactive network tissue, Neural stem cell paracrine effect, Single cell rnaseq analysis

## Abstract

Spinal cord injury (SCI) results in sensory and motor dysfunction, with neuronal death, circuit disruption, and the inhibitory microenvironment serving as key limitations to effective treatment. In this study, we developed a neuroactive network tissue for SCI repair by immobilizing dual recombinant growth factors based on biomimetic mussel adhesive units onto an oriented electrospun nanofiber scaffold, and seeding neural stem cells (NSCs) onto the scaffold. This dual-factor system continuously stimulates and enhances the paracrine function of NSCs, promoting repair of the injury site. In the early stages, the neurorepair coating amplifies the paracrine effects of NSCs, alleviating oxidative stress and inflammation while inhibiting neuronal cell death. In the later stages, it facilitates neurogenesis, axon growth, and neural circuit restoration. Single-cell RNA sequencing further reveals that the treatment reduces immune cell activation, promotes the survival of neurons and oligodendrocytes, sequentially and multidimensionally improves the local microenvironment, and enhances tissue regeneration. Both in vitro and in vivo experiments confirms that the neural active network effectively reshapes the immune environment at the injury site, boosting cell differentiation and repair, and thus providing a comprehensive strategy for tissue regeneration.

## Graphical abstract

**Figure:**
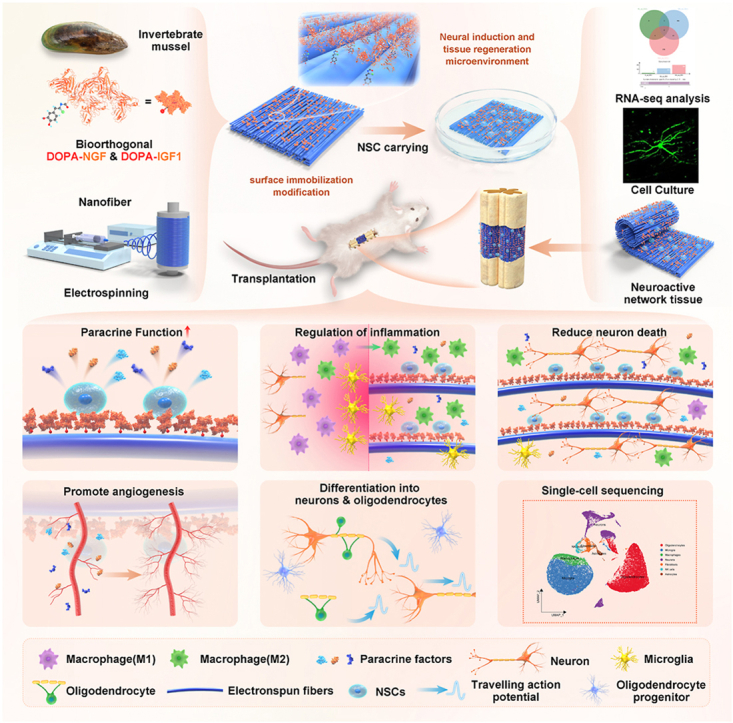

## 1 Introduction

Spinal cord injury (SCI) is a severe condition that results in long-term sensory and motor impairments, affecting hundreds of thousands of individuals worldwide each year and imposing a significant burden on society and patients' families [1]. SCI can be classified into primary and secondary injuries. The primary injury results from direct, irreversible mechanical trauma, whereas the secondary injury involves complex pathological processes that exacerbate damage and inhibit neural regeneration through a cascade of molecular events [2]. Although recent research has focused on therapeutic targets during the secondary injury phase, treatment outcomes remain limited due to neuronal loss, disruption of neural circuits, and the presence of a complex inhibitory microenvironment [3].

Neural tissue engineering has emerged as a highly promising therapeutic strategy, showing significant potential for the repair of SCIs [4]. The use of bioengineered degradable biomaterials in conjunction with neural stem cells (NSCs) creates a favorable microenvironment that supports the growth and proliferation of seed cells, laying the foundation for effective tissue regeneration. Simultaneously, bioactive materials improve the inhibitory local microenvironment and regulate cellular behavior, significantly enhancing cell survival and bioactivity, ultimately promoting neural tissue repair a), [5]. Electrospinning technology has been widely applied in neural tissue engineering, enabling the fabrication of nanofiber scaffolds that mimic the extracellular matrix to guide directional growth of neural cells and regulate cellular behavior [6]. Furthermore, bioactive modifications with growth factors, antibodies, or drugs can enhance the scaffold's bioactivity and effectively modulate the local microenvironment [7]. NSCs, as essential seed cells, possess paracrine functions and multipotent differentiation capabilities. They can secrete neurotrophic factors, promote neuroprotection and immunomodulation, and differentiate into neurons and oligodendrocytes, facilitating neural circuit reconstruction [8]. However, the inhibitory microenvironment following SCI significantly suppresses the bioactivity of NSCs and their differentiation into neurons [9]. Although preconditioning NSCs with growth factors or co-transplanting them to promote survival and differentiation into new neurons has shown potential for neural repair and axonal growth, the therapeutic efficacy is limited by growth factor diffusion and dynamic changes in the microenvironment a), [10]. To address these challenges, the application of bioactive modified interfaces is particularly important, as they provide sustained biological stimulation, enhance cell survival, and improve SCI repair outcomes [11]. Although our previous studies show that growth factor coatings enhance the paracrine effects of stem cells, research on growth factor immobilization in SCI remains limited, and the optimal modification concentration is still undetermined [12].

**Scheme 1:**
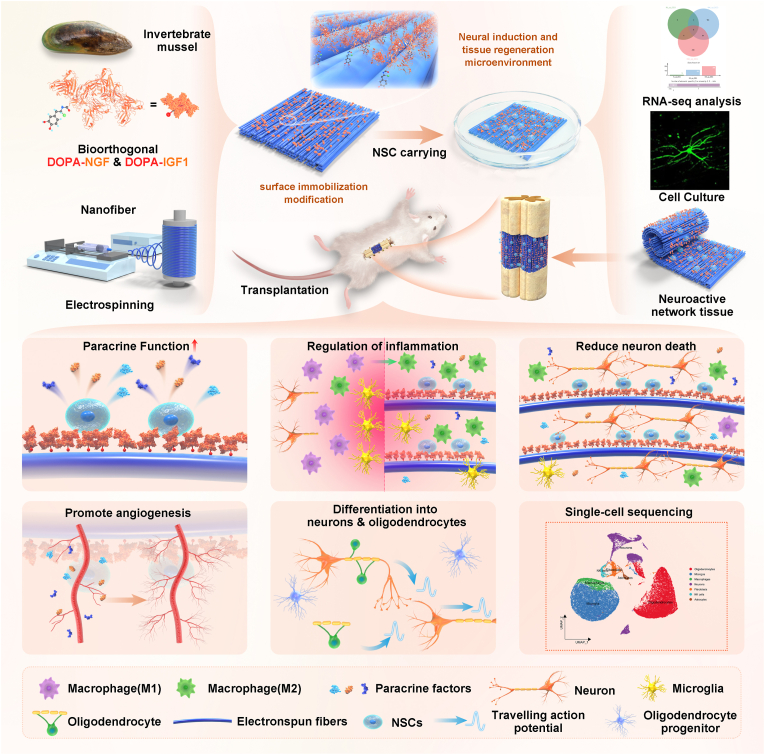
Schematic illustration of the construction and transplantation of neuroactive network tissue for SCI treatment. In the early stages, the neuroactive network tissue amplified the paracrine effects of NSCs, alleviating oxidative stress and inflammation while inhibiting neuronal cell death. In the later stages, it facilitated neurogenesis, axon growth, vascular regeneration and neural circuit restoration. By sequentially and multidimensionally optimizing the regenerative microenvironment, this approach significantly enhanced SCI repair outcomes.

We pioneered the concept of growth factor density to prevent the loss of freely diffusing growth factors. By immobilizing recombinant growth factors functionalized with bioinspired mussel adhesive motifs onto the surface of oriented electrospun nanofiber scaffolds, we engineered a neuroregenerative bioactive coating that enhances growth factor retention and promotes tissue repair. When combined with NSCs, this system forms neuroactive network tissue, presenting a promising strategy for SCI repair. This neuroactive network tissue not only exhibits robust stem cell paracrine effects but also demonstrates highly efficient neuronal differentiation capabilities. The sustained stimulation provided by the dual-factor neuroregenerative bioactive coating profoundly boosts the paracrine function of NSCs, amplifying their regenerative potential. In the early phase, it modulates the inhibitory microenvironment at the injury site, attenuates programmed cell death, and facilitates the generation of new neurons and axonal outgrowth, ultimately promoting tissue regeneration. We utilized bulk and single-cell RNA sequencing (scRNA Seq) to elucidate the molecular mechanisms by which the neuroactive network tissue exerts its protective effects and modulates immune responses in the early stages of SCI repair. These findings uncovered dynamic cellular reprogramming, revealing the diverse functional and phenotypic characteristics of cells within the injury tissues microenvironment, which play a crucial role in the regenerative process. This discovery highlights key cell subtypes involved in tissue regeneration, uncovers potential therapeutic targets, and provides crucial insights for developing advanced strategies in SCI treatment (Scheme 1).

## 2 Result

### 2.1 Preparation and bioactivity evaluation of bioinspired immobilized growth factor coatings

**Fig. 1:**
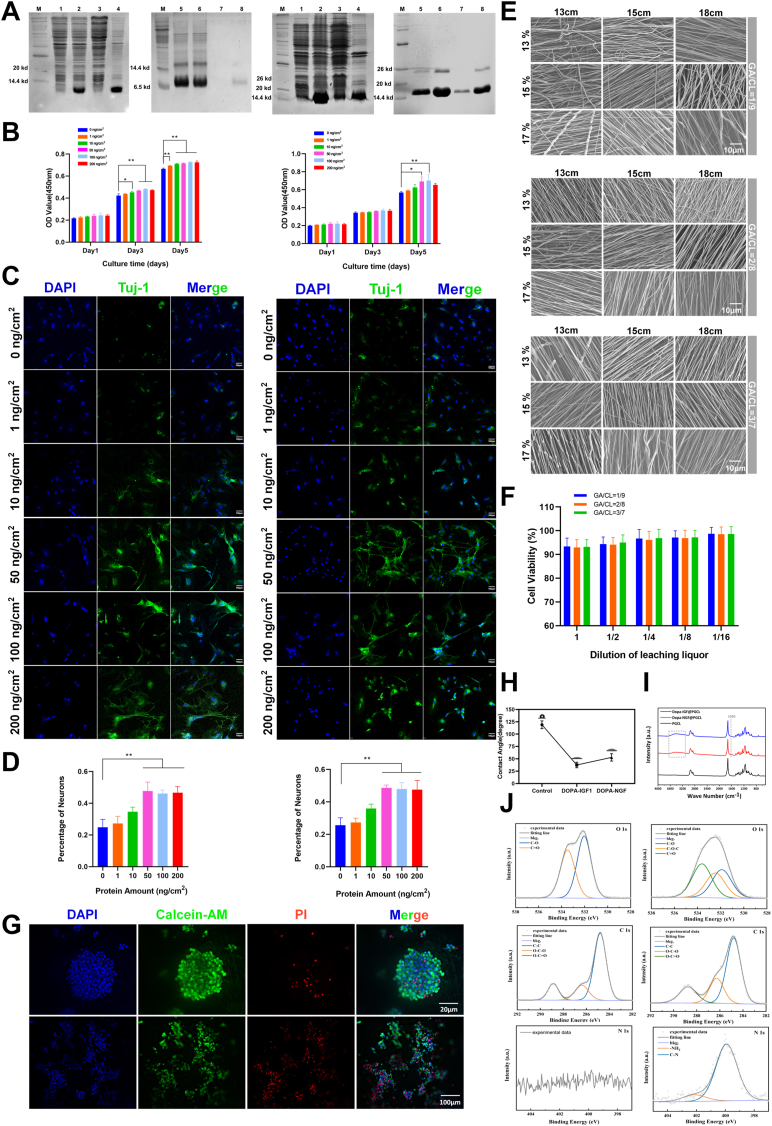
Preparation and bioactivity evaluation of immobilized recombinant growth factors and the construction of the neuroregenerative coating fiber network scaffold. (A) SDS-PAGE gel showed the preparation process of immobilized recombinant growth factors D-IGF1 and D-NGF including: marker (M), pre-induction (1), post-induction (2), supernatant after centrifugation (3), pellet after centrifugation (4), refolded protein solution (5), flow-through liquid (6), wash liquid (7), elution liquid (8). (B) The effect of different densities of D-IGF1 and D-NGF on NSC proliferation. (C–D) The effect of different densities of D-IGF1 and D-NGF on neural stem cell differentiation. Scale bars, 100 μm (E) SEM images of the surface morphology of fiber scaffolds prepared under different process parameters. Scale bars, 10 μm (F–G) Biocompatibility testing of the scaffold. (H) Contact angle analysis. (I) FT-IR analysis. (J) XPS analysis. (Data are shown as the mean ± SD, n = 3, ∗p < 0.05,∗∗p < 0.01).

IGF-1 and NGF are essential growth factors that play pivotal roles in promoting neural tissue repair and regeneration [13]. To immobilize these factors onto the surface of polymer-based oriented network structures and create fiber network scaffolds with neuroregenerative potential, we employed bioorthogonal chemistry. This approach integrates recombinant DNA technology with enzymatic reactions involving non-canonical amino acids, replicating the DOPA motifs found in mussel adhesive proteins. Specifically, we introduced a pentapeptide tag (Tyr-Lys-Tyr-Lys-Tyr, YKYKY) at the C-terminus of IGF-1 and NGF. This tag was enzymatically modified by tyrosine hydroxylase to produce a DOPA-enriched pentapeptide (DOPA-Lys-DOPA-Lys-DOPA, XKXKX; X = DOPA), effectively mimicking the adhesive properties of mussel-inspired proteins. This modification preserved the bioactivity and tertiary structure of the growth factors, enabling the fabrication of bioinspired immobilized recombinant growth factors linked by DOPA adhesive motifs. To assess the feasibility of this strategy, recombinant proteins (YKYKY-IGF1 and YKYKY-NGF) were expressed in Escherichia coli BL21 followed by characterization using SDS-PAGE and Western blot analysis. SDS-PAGE analysis revealed that Y-IGF1 and Y-NGF exhibited molecular weights of approximately 9.5 kDa and 16.5 kDa, respectively, slightly exceeding those of the native proteins (7.4 kDa and 14.5 kDa). These findings align with the predicted molecular weights following the addition of the pentapeptide and His-tag (Fig. 1A). Western blot analysis further confirmed that the engineered modifications did not affect the protein structure, with antibody reactivity similar to that of commercial proteins (Fig. S1).

The bioinspired immobilized recombinant proteins adhered strongly to the surface of biomaterials, forming a bioactive coating that facilitated sustained biological activity. This coating significantly enhanced surface hydrophilicity, promoting cell adhesion and tissue growth. In comparison to traditional soluble growth factors, immobilized growth factors offer precise dosage control and substantially improve protein utilization efficiency [14]. Building on previous studies, we assessed the adhesion efficiency and stability of bioinspired immobilized recombinant proteins on polymer surfaces [15]. Experimental results demonstrated that, within the range of 0–100 ng, adhesion efficiency increased proportionally with the protein dosage. Beyond 100 ng, adhesion efficiency plateaued, suggesting near-complete immobilization of the proteins on the material surface. In addition, we examined the stability of recombinant proteins at different application concentrations. The results indicated that at higher application concentrations, the recombinant proteins exhibited lower release rates and enhanced stability, enabling sustained bioactivity on the material surface and biological effects (Fig. S2). We propose that this effect arises from crosslinking interactions between DOPA molecules, which form densely crosslinked networks at higher concentrations, thereby enhancing protein adhesion to the material surface [11c].

**Fig. 2:**
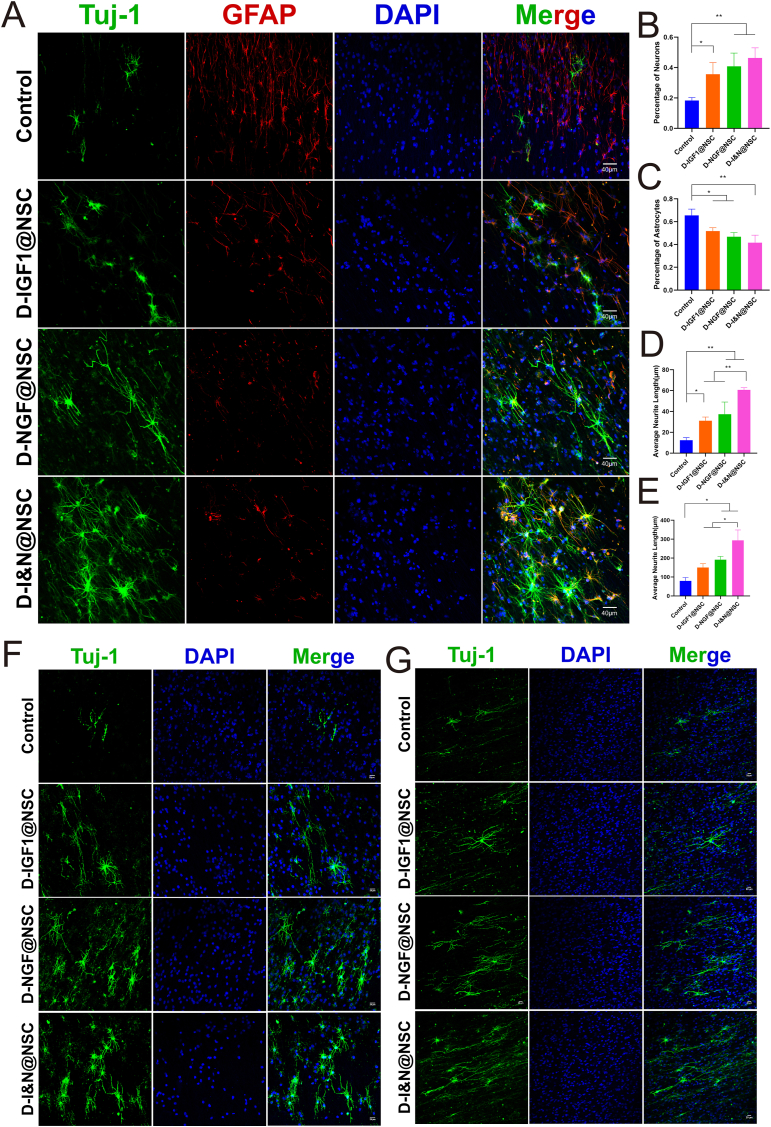
Construction of neuroactive network tissue and characterization of neuroinductive activity. (A) Immunofluorescence staining of Tuj-1 (green) and GFAP (red). Scale bars, 40 μm (B–C) Quantitative analysis of neurons and astrocytes. (D–E) Quantitative analysis of axonal length in neuroactive network tissue at 7 and 14 Days (F–G) Immunofluorescence staining of axonal growth in neuroactive network tissue at 7 and 14 days. Scale bars, 20 μm (Data are shown as the mean ± SD, n = 3, ∗p < 0.05,∗∗p < 0.01). (For interpretation of the references to color in this figure legend, the reader is referred to the Web version of this article.)

The following experiments were used to assess the bioactivity of bioinspired immobilized recombinant proteins. NSCs were isolated from the hippocampi of fetal rat brains and identified using immunofluorescence staining. After 48 h of culture, NSCs exhibited a tendency to aggregate growth, forming neurospheres, with positive expression of nestin (Fig. S3). Compared to traditional soluble growth factors, immobilized recombinant growth factors exhibit enhanced biological effects [12]. The regulatory impact of immobilized proteins on NSC proliferation and differentiation were evaluated using the CCK-8 assay and immunofluorescence staining. Cellular behavior was quantitatively assessed based on growth factor density, defined as the amount of protein per unit area. Both immobilized recombinant proteins promoted cell proliferation across a range of growth factor densities (DOPA-IGF1 and DOPA-NGF at 0, 1, 10, 50, 100, and 200 ng cm^−2^), with DOPA-IGF1 demonstrating a more pronounced effect. Cell proliferation enhancement plateaued at growth factor densities above 50 ng cm^−2^, indicating that excessively high densities may have detrimental effects (Fig. 2B). NSCs were cultured at various growth factor densities, and their differentiation into newly formed neurons was evaluated on day 7 using immunofluorescence staining (Fig. 2C–D). Differentiation into neurons was more pronounced in the DOPA-NGF group than in the DOPA-IGF1 group, with the effect increasing as growth factor density rose. However, when the growth factor density exceeded 50 ng cm^−2^, the number of newly formed neurons plateaued, indicating that higher concentrations do not further enhance biological activity.

In summary, a growth factor density of 50 ng cm^−2^ exhibited the highest biological activity for both recombinant proteins, and was therefore chosen for preparing neuroregenerative bioactive coatings in subsequent experiments.

### 2.2 Preparation and characterization of neuroregenerative bioactive coating fiber network scaffolds

We utilized electrospinning technology to fabricate oriented nanofiber network scaffolds with specialized topological structures, designed to replicate the properties of the extracellular matrix and support the directional growth of neuronal axons. To optimize electrospinning parameters, an orthogonal experimental design was employed, varying solution concentrations (13 wt%, 15 wt%, and 17 wt%) and spinning distances (13 cm, 15 cm, and 18 cm). Using three polymer blends with varying GA:CL ratios (10:90, 20:80, and 30:70), we successfully fabricated 27 distinct oriented scaffolds (Fig. 2E). The results showed that increasing the spinning distance reduced fiber diameter, producing smoother, more uniform fibers with improved alignment. Higher solution concentrations led to larger fiber diameters and better alignment but also promoted the formation of nodules and bead-like structures (Fig. S4). To achieve optimal surface morphology and structural properties for cell adhesion and axonal growth, a spinning distance of 18 cm and a solution concentration of 15 wt% were identified as the optimal parameters for subsequent experiments. The mechanical properties of materials with varying GA:CL ratios were then assessed to ensure their suitability for withstanding mechanical stresses associated with SCI (Fig. S5A–E). The results indicated that materials with GA:CL ratios of 10:90 and 20:80 exhibited superior mechanical properties, while the GA:CL = 30:70 formulation showed inferior mechanical performance due to poor solubility and increased structural irregularities. A moderate increase in GA content enhanced the material's degradability, making it more suitable for tissue regeneration and repair applications [16]. Therefore, the GA:CL ratio of 20:80 was selected for subsequent experiments.

The biocompatibility of the oriented nanofiber scaffolds, prepared with GA:CL ratios of 10:90, 20:80, and 30:70, was evaluated using material extraction assays and live/dead cell staining. The results showed that cell viability exceeded 90 % in both undiluted and diluted extraction solutions. Moreover, cell viability further increased with higher dilution of the extraction solution (Fig. 2F). NSCs adhered to, survived on, and proliferated within the nanofiber scaffolds, forming neurospheres, with the majority of cells remaining viable. Low-magnification microscopy revealed a multilayered distribution of cells within the scaffold. The scaffold's highly porous structure, extensive surface area, and high porosity created a favorable microenvironment that promoted cellular adhesion and proliferation (Fig. 2G).

To enhance the neuroregenerative potential and axonal growth-promoting activity of the oriented network scaffolds, as well as to improve their cell adhesion properties and ability to regulate cellular behavior, we functionalized the scaffold surfaces with biospired immobilized recombinant proteins to create bioactive coatings for neural repair. The surface modifications of the scaffolds were characterized using Fourier transform infrared spectroscopy (FT-IR), X-ray photoelectron spectroscopy (XPS), and contact angle measurements. The results showed a significant increase in the hydrophilicity of the modified scaffolds, which facilitated neural cell attachment, adhesion, and growth (Fig. 2H). FT-IR analysis revealed the emergence of an absorption peak at 1620 cm^−1^ in the modified nanofiber network scaffolds, corresponding to the C=O stretching vibration of amide bonds in the immobilized growth factors, absent in the unmodified scaffolds. Characteristic peaks for N–H and O–H stretching vibrations were observed in the 3200∼3600 cm^−1^ range, confirming the successful immobilization of growth factors on the scaffold surface. Additionally, the FT-IR spectra of DOPA-IGF1 and DOPA-NGF functionalized samples exhibited similar patterns, reflecting their chemical structural similarity (Fig. 2I). XPS analysis further confirmed the successful surface modification of the scaffolds. In the PGCL group, two characteristic O 1s peaks were detected at 533.5 eV and 532 eV, corresponding to C=O and C–O bonds in the PCL and PGA segments, respectively. After growth factor functionalization, the intensity of the C=O peak significantly increased, indicating a higher C=O bond content derived from amino acids. Additionally, a characteristic peak corresponding to ether oxygen bonds C–O–C was observed at 532.5 eV, attributed to the surface-immobilized growth factor proteins. The C 1s spectrum revealed three characteristic peaks in the PGCL group at 288.9, 286.4, and 284.8 eV, corresponding to O–C=O, O–C–O, and C–C bonds, respectively. After growth factor functionalization, the intensity of the O–C–O peak increased significantly, indicating a higher carboxyl group content derived from amino acids. In the N 1s spectrum, no distinct peaks were observed in the unmodified PGCL group. However, after growth factor functionalization, characteristic peaks corresponding to 399.9 eV (C–N) and 402.2 eV (–NH_2_) appeared, further confirming the successful immobilization of the growth factors (Fig. 2J).

### 2.3 Construction of neuroactive network tissue and characterization of neuroinductive, neuroprotective, and immunomodulatory functions

**Fig. 3:**
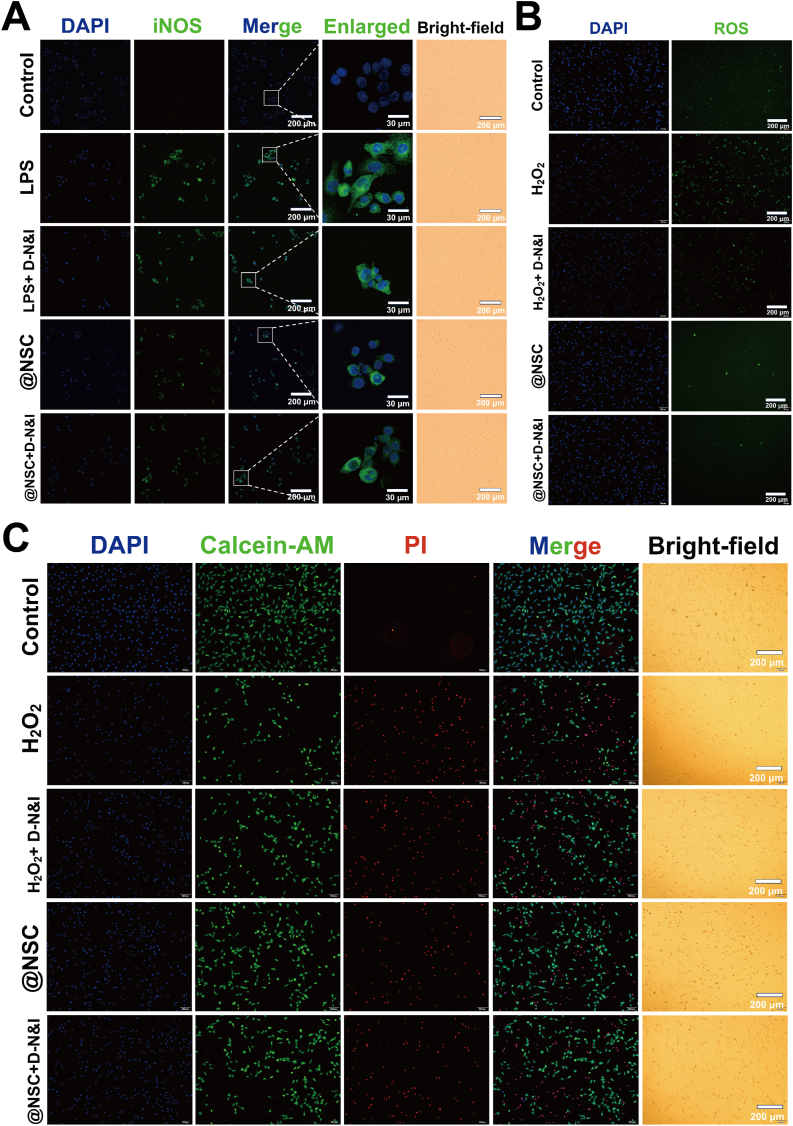
Protective effects of the dual-factor neuroactive network tissue on cells and its modulation of the inflammatory response. (A) Immunofluorescence staining of iNOS in RAW 264.7 macrophages co-cultured with different treatment groups in the Transwell system. Scale bars, 30 μm, 200 μm (B) DCFH-DA staining to assess intracellular ROS levels in HT22 cells co-cultured with different treatment groups in the Transwell system. Scale bars, 200 μm (C) Calcein-AM and propidium iodide staining of HT22 cells co-cultured in the Transwell system under different treatment groups. Scale bars, 200 μm.

By integrating recombinant DNA technology and enzymatic reactions with non-canonical amino acids, we engineered oriented nanofiber network scaffolds functionalized with neuroregenerative bioactive coatings. Combined with NSCs, these scaffolds formed neuroactive network tissue with neuroinductive, tissue-regenerative, and immunomodulatory properties. Immunofluorescence staining showed that the D-NGF coating better promoted NSCs differentiation into neurons than D-IGF1 coating. The dual-factor neuroregenerative bioactive coating significantly enhanced neuroinduction and axonal growth, promoted the production of new neurons, and reduced astrocyte proliferation. These results have important implications for improving neural repair and mitigating glial scar formation after SCI (Fig. 2A–C). Cultivation experiments at 7 and 14 days demonstrated that the dual-factor-coated neuroactive network tissue exhibited enhanced axonal guidance capabilities and facilitated the formation of tight connections between newly generated neurons (Fig. 3D–G).

Previous investigations have shown that immobilized growth factors significantly enhance the paracrine activity of mesenchymal stem cells upon direct interaction. Building on this premise, we hypothesize that fiber network scaffolds functionalized with dual-factor neuroregenerative bioactive coatings and seeded with NSCs can amplify the paracrine activity of NSCs in the early phases of SCI, promoting the development of neuroactive network tissue. By modulating the paracrine activity of NSCs, the network facilitates the release of bioactive molecules, such as growth factors, cytokines, and regulatory peptides. These molecules, in combination with the regenerative coating, establish a microenvironment that promotes tissue repair. This environment helps mitigates programmed cell death, enhances cellular resistance to oxidative stress, and regulates the local immune response, thereby supporting neural remodeling and tissue regeneration following SCI. The following experiments were designed and conducted to test this hypothesis.

#### 2.3.1 Oxidative stress response

In the experiment, H2O2-treated HT22 cells showed a significant increase in.ROS production, triggering an oxidative stress response. As shown in Fig. 3B The dual-factor bioactive coating effectively reduced ROS production. Furthermore, HT22 cells co-cultured with neuroactive network tissue exhibited significantly enhanced resistance to oxidative stress. H2O2 induces an increase in ROS levels in HT22 cells, leading to a reduction in mitochondrial membrane potential, which subsequently triggers mitochondrial dysfunction and apoptosis [17]. The results showed that H2O2 treatment led to a decrease in mitochondrial membrane potential and reduced cell viability in HT22 cells. However, these effects were significantly mitigated when the cells were co-cultured with neuroactive network tissue (Fig. S6).

#### 2.3.2 Immunomodulation of inflammatory response

**Fig. 4:**
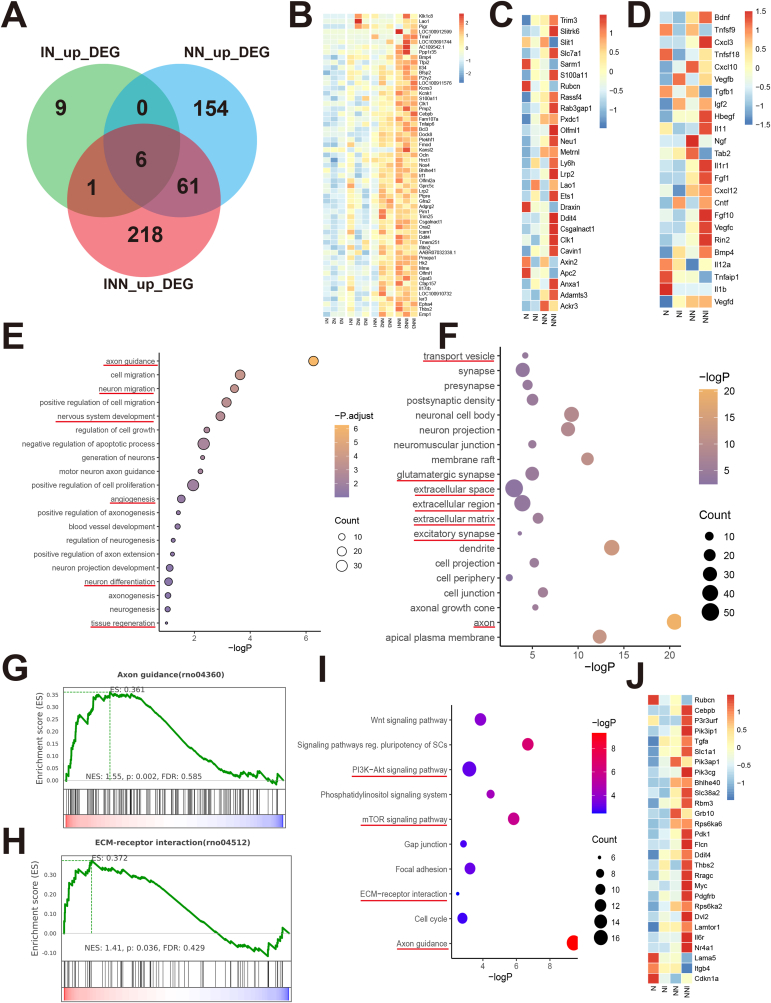
Bulk RNA-Seq analysis revealing the regulatory effects of dual-factor and single-factor neuroregenerative coatings on NSCs. (A) Venn diagram showing the differential gene expression in neural stem cells treated with D-IGF1, D-NGF, and D-IGF1 & D-NGF neuroregenerative coatings. (B) Heatmap showing gene expression regulation of NSCs by different single-factor and dual-factor neuroregenerative coatings. (C) Heatmap of gene expression related to neurogenesis, axonal growth, angiogenesis, and tissue regeneration in NSCs under different treatments. (D) Heatmap of gene expression related to paracrine proteins and cytokines in NSCs under different treatments. (E) GO analysis of the biological processes enriched by differentially expressed genes. (F) GO analysis of the cellular component categories enriched by differentially expressed genes. (G–H) GSEA Analysis reveals significant activation of genes related to axonal growth and extracellular matrix receptor interactions. (I) KEGG pathway analysis reveals enriched pathways and their significance among differentially expressed genes. (J) Heatmap of gene expression related to the PI3K-Akt-mTOR pathway in NSCs under different treatments.

Macrophages were stimulated with LPS to mimic the inflammatory response following SCI, enabling the assessment of the immunoregulatory effects of the dual-factor coating and neuroactive network tissue. Immunofluorescence staining revealed an upregulation of iNOS expression and a transition to an elongated, spindle-like morphology, which are indicative of LPS-induced polarization of M0 macrophages toward the pro-inflammatory M1 subtype (Fig. 4A). The experimental results demonstrated that the dual-factor coating effectively suppressed iNOS expression, while the neuroactive network tissue exhibited enhanced immunomodulatory effects. These tissues significantly reduced iNOS expression and minimized morphological changes in macrophages.

#### 2.3.3 Programmed cell death

Live/dead cell staining further confirmed that neuroactive network tissue significantly attenuated programmed cell death under simulated SCI conditions. These findings suggest that neuroactive network tissue promote cell survival through multiple mechanisms, including enhanced antioxidant defenses, reduced ROS production, and preservation of mitochondrial function (Fig. 4C).

### 2.4 RNA-seq reveals dual-factor active coatings promote neural tissue regeneration by regulating the gene expression of NSCs

**Fig. 5:**
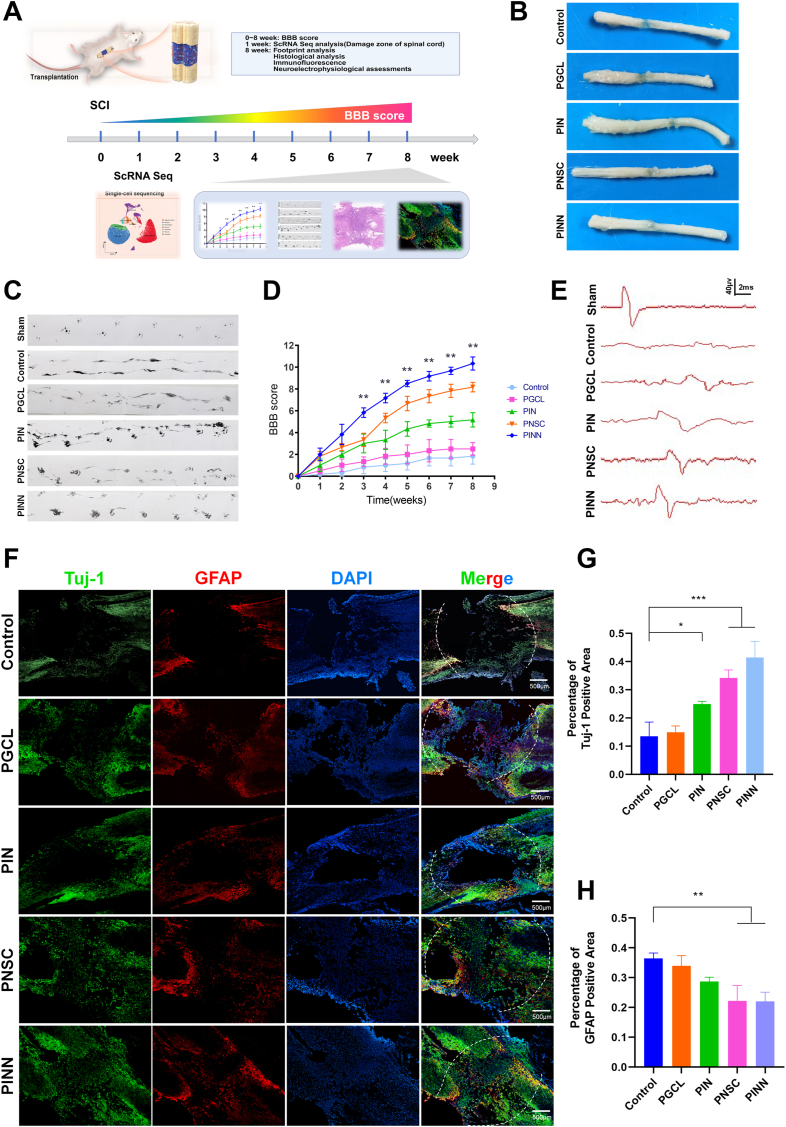
Neuroactive network tissue promotes tissue regeneration and functional recovery in SCI rats. (A) Experimental timeline of SCI model. (B) Images of spinal cord tissue at 8 weeks post-surgery. (C) Footprint analysis images at 8 weeks post-surgery (D) BBB score. (E) Representative images of MEPs in the different groups at 8 weeks post-surgery. (F) Immunofluorescence staining of Tuj-1 (green) and GFAP (red) to observe neurons and astrocytes in the injured spinal cord tissue of different groups. Scale bars, 500 μm (G–H) Quantitative analysis of Tuj-1 positive area and GFAP positive area in the injury region of different groups. (Data are shown as the mean ± SD, n = 3, ∗p < 0.05,∗∗p < 0.01). (For interpretation of the references to color in this figure legend, the reader is referred to the Web version of this article.)

Preliminary experiments have demonstrated that the fiber network scaffold, infused with a dual-factor neural repair active coating, can seamlessly integrate with NSCs to form a neuroactive network tissue. This synergy fosters a regenerative microenvironment conducive to tissue healing in the early stages. We believe that the amplification and fine-tuning of paracrine signaling from the seed cells, coupled with the precise regulation of cell differentiation, are pivotal drivers of this regenerative process [6a]. To investigate the regulatory effects of bioactive coatings on the gene expression of NSCs, we performed transcriptome sequencing analysis on the NSCs cultured for 3 days on the following scaffolds: the pure PGCL scaffold (N), the DOPA-IGF1 coated scaffold (IN), the DOPA-NGF coated scaffold (NN), and the dual-factor D-IGF1&D-NGF bioactive coated scaffold (INN). From the DEG analysis, we found that the number of upregulated DEGs in the NN group was significantly higher compared to the IN group, suggesting that the D-NGF coating exerts a stronger regulatory effect on NSCs than the D-IGF1 coating. The dual-factor coating demonstrated a clear synergistic effect, significantly enhancing both the expression and the number of upregulated DEGs (Fig. 4A–B). Dual-factor coating promotes neural tissue remodeling, inhibiting oxidative stress and apoptotic responses. Upon analyzing the expression of genes related to neurodevelopment and tissue regeneration, we found that under continuous stimulation by the dual-factor coating, genes associated with neuronal development, synapse formation, axon regeneration, and angiogenesis were significantly upregulated, while genes with negative regulatory effects were downregulated (Fig. 5C). Transwell experiments vividly demonstrated the pivotal role of paracrine signaling from NSCs. Gene expression analysis of paracrine factors demonstrated that the dual-factor bioactive coating significantly enhanced the expression of genes associated with neuronal development and survival, angiogenesis, tissue repair, cellular metabolism, and migration, while concurrently downregulating genes related to pro-inflammatory cytokines (Fig. 5D). These findings suggest that neuroactive network tissue enhance local cell survival, accelerate tissue repair, and modulate immune responses through precise regulation of the paracrine signaling pathways of NSCs.

We used functional annotation of the DEGs in the dual-factor group and found that these genes are primarily involved in biological processes such as axon growth, neurogenesis, neuronal differentiation, cell proliferation, angiogenesis, and tissue regeneration. Enrichment analysis of cellular components revealed that the DEGs are predominantly localized to the extracellular matrix, extracellular transport vesicles, extracellular space, neuronal axons, dendrites, and synapses (Fig. 5E–F). We further conducted GSEA analysis on genes associated with axon growth and extracellular matrix-receptor interactions, revealing a striking activation of both processes (Fig. 5G–H). These results provide compelling evidence that dual-factor stimulation dramatically enhances the paracrine effects of NSCs, as well as their capacity for neuronal differentiation.

Furthermore, KEGG analysis indicated that the dual-factor active coating significantly activates the PI3K-Akt-mTOR pathway (Fig. 5I–J). We propose that the dual-factor coating enhances the paracrine effects of NSCs in the early stages by activating the PI3K-Akt-mTOR pathway. This activation helps establish a favorable tissue microenvironment, which, in turn, promotes neuronal differentiation and axon growth, ultimately facilitating tissue repair.

### 2.5 Therapeutic effects of neuroactive network tissue transplantation on SCI repair

We established a complete transection SCI model in rats to assess the therapeutic potential of neuroactive network tissue, engineered with dual-factor neuroregenerative-coated fiber scaffolds and NSCs. After 8 weeks, all experimental groups showed varying degrees of recovery (Fig. 5A–B). In the Control group, a gap remained at the injury site. The PGCL group showed persistent scar tissue and cavities. The PIN group exhibited moderate repair. while the PNSC group had reduced cavity size. Notably, the PINN group, transplanted with neuroactive network tissue, showed the most significant repair, with a marked decrease in cavity size and scar tissue.

**Fig. 6:**
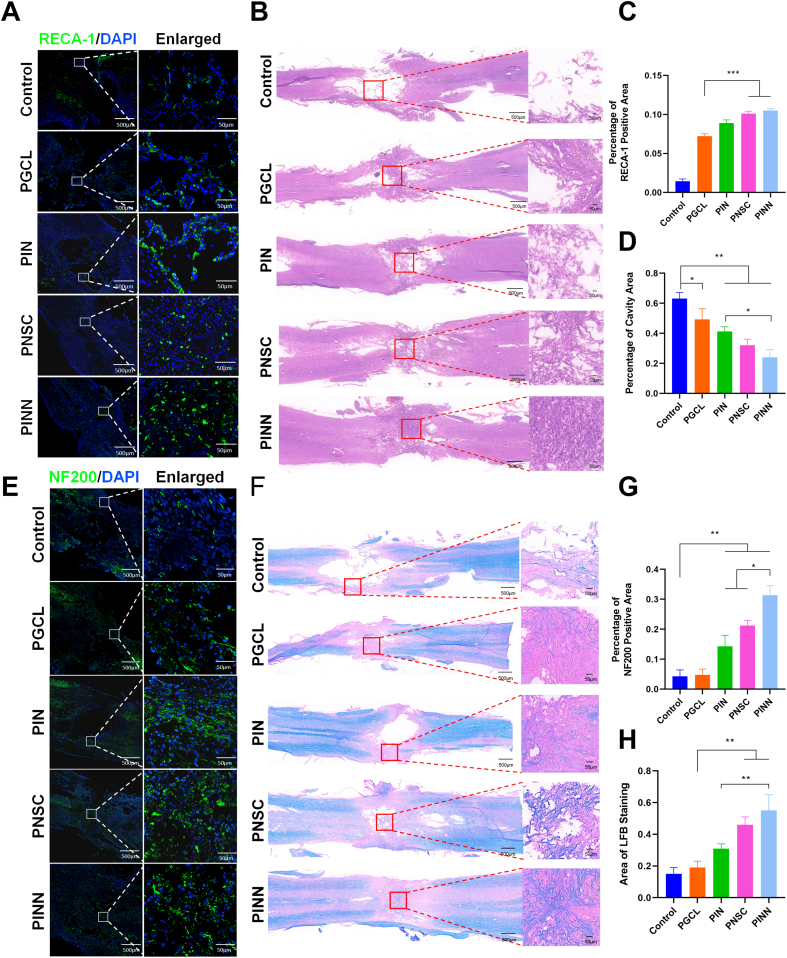
Neuroactive network tissue promotes tissue regeneration in SCI rats. (A) Immunofluorescence staining of RECA-1 to observe blood vessel regeneration in the injured spinal cord tissue of different groups. Scale bars, 500 μm, 50 μm (B) HE staining of injured spinal cord tissue in different groups. Scale bars, 500 μm, 50 μm (C) Quantitative analysis of vascular regeneration in the injury region of different groups. (D) Quantitative analysis of the cavity area from HE staining in the injury region of different groups. (E) Immunofluorescence staining of NF200 to observe axonal regeneration in the injured spinal cord tissue of different groups. Scale bars, 500 μm, 50 μm (F) LFB staining of injured spinal cord tissue in different groups. Scale bars, 500 μm, 50 μm (G) Quantitative analysis of axonal regeneration in the injury region of different groups. (H) Quantitative analysis of the myelin area from LFB staining in the injury region of different groups. (Data are shown as the mean ± SD, n = 3, ∗p < 0.05,∗∗p < 0.01).

**Fig. 7:**
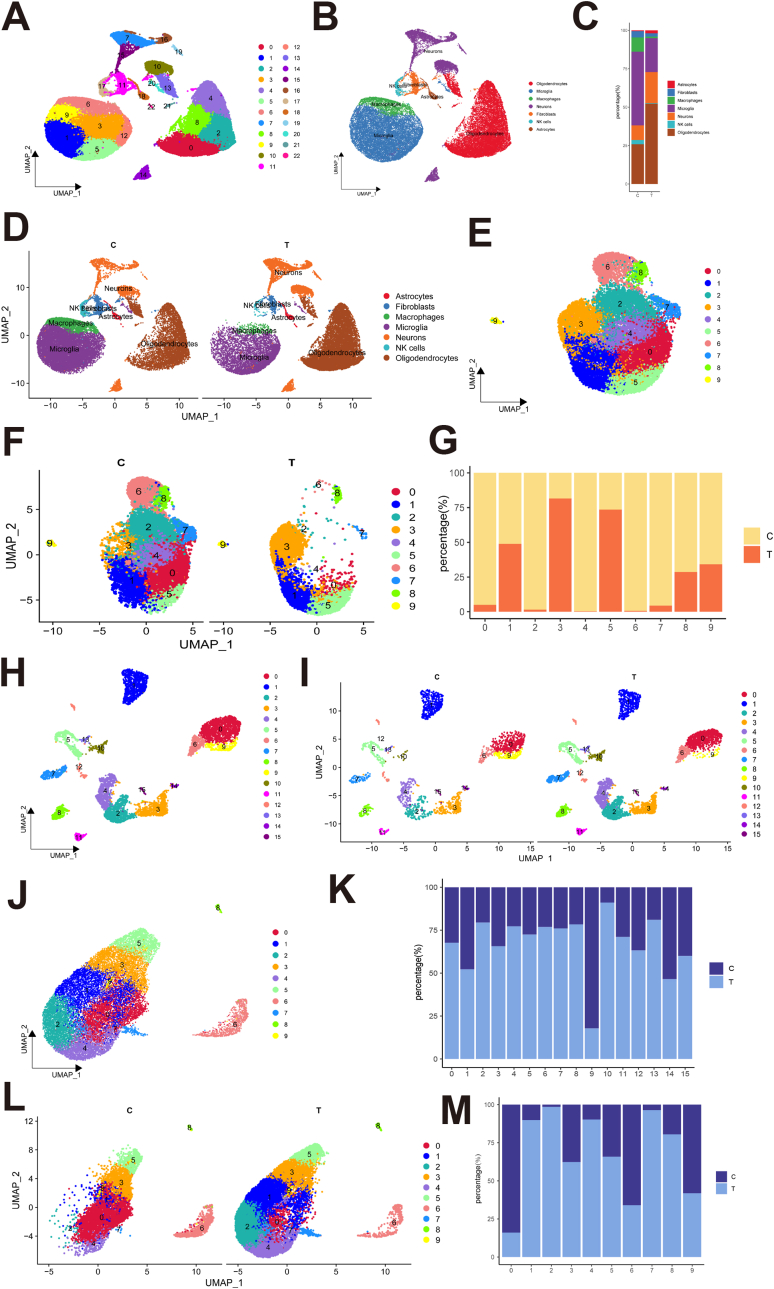
ScRNA seq analysis revealed that the transplantation of the neural active network constructs a regenerative microenvironment, providing protective and modulatory effects on local tissue cells during the early stages of SCI. (A–B) The UMAP plot revealed cellular diversity by identifying 23 distinct cell clusters, each represented in a unique color. The general identities of these clusters are outlined on the right side. The UMAP plot, colored according to cell types, highlights the cellular diversity. (C) Percentage of each cell type in spinal cord tissue of negative control and treatment groups. (D) The UMAP plot colored by cell types in spinal cord tissue of negative control and treatment groups. (E) the UMAP plot revealed the distribution of 10 microglia/macrophage subtypes based on spinal cord tissue. (F) The UMAP plot revealed cellular heterogeneity, with 10 distinct cell subtypes identified and color-coded within the spinal cord tissue of negative control and treatment groups. (G) The proportion of negative control and treatment groups within each microglia/macrophage subtype. (H) the UMAP plot revealed the distribution of 16 neurons subtypes based on spinal cord tissue. (I) The UMAP plot revealed cellular heterogeneity, with 16 distinct neurons subtypes identified and color-coded within the spinal cord tissue of negative control and treatment groups. (J) the UMAP plot revealed the distribution of 10 oligodendrocytes subtypes based on spinal cord tissue. (K) The proportion of negative control and treatment groups within each neurons subtype. (L) The UMAP plot revealed cellular heterogeneity, with 10 distinct oligodendrocytes subtypes identified and color-coded within the spinal cord tissue of negative control and treatment groups. (M) The proportion of negative control and treatment groups within each oligodendrocyte subtype. (For interpretation of the references to color in this figure legend, the reader is referred to the Web version of this article.)

Hematoxylin-eosin (H&E) staining showed superior tissue repair in the PINN group, with no visible cavities. The regenerated neural tissue integrated seamlessly with the surrounding native tissue, accompanied by reduced local inflammation (Fig. 6B and D). Luxol Fast Blue (LFB) staining revealed a progressive increase in myelin density and myelin-positive areas. Notably, the PINN group had the highest number of regenerated myelinated fibers, forming an interconnected network with the surrounding fibrous tissue (Fig. 7F and H).

Immunofluorescence staining was performed to assess neurogenesis, astrocyte activation, axonal regeneration, vascular formation, and inflammatory responses at the injury site in SCI rats. Newly formed neurons and astrocytes were identified by Tuj-1 and GFAP staining, respectively (Fig. 6F and 6G–H). The Control and PGCL groups showed sparse Tuj-1-positive neurons, more GFAP-positive astrocytes, and prominent vacuoles at the injury site. The PIN group had more newly formed neurons but still displayed a large cavity area. Compared to the control, both the PNSC and PINN groups had more newly generated neurons. The PINN group showed the highest density of Tuj-1-positive neurons, less astrocyte proliferation, and better tissue integration. NF200 staining was employed to assess axonal regeneration (Fig. 7E and G). The PINN group showed extensive axonal growth with highly interconnected structures, leading to the most comprehensive tissue repair. Vascular regeneration, marked by RECA-1 staining (Fig. 7A and C), was most prominent in the PINN group. Inflammatory responses, assessed by IBA1 and CD68 staining, revealed the lowest levels of inflammatory cell activation and minimal localized inflammation in the PINN group, highlighting the superior immunomodulatory properties of the neuroactive network tissue that support effective tissue repair (Fig. S8A–B). In addition, at 4 weeks post-implantation, H&E staining revealed no histopathological alterations in the heart, liver, spleen, lungs, or kidneys across all groups, indicating good biocompatibility of the scaffolds (Fig. S7A). Serial photographs of subcutaneous implants taken at predetermined time points demonstrated favorable degradation profiles, consistent with the requirements for tissue repair (Fig. S7B).

Motor function recovery was evaluated using BBB scoring and footprint analysis. Rats in the PINN group, which received neuroactive network tissue transplants, showed the highest BBB scores. Significant improvements over the Control group were observed by the third postoperative week and persisted throughout the assessment period (Fig. 6D). Footprint analysis showed that rats in the PINN group had gait patterns similar to the sham-operated group, with fewer toe drag marks and smaller paw rotation angles, indicating better paw support and improved locomotor coordination (Fig. 6C). In contrast, the Control and PGCL groups showed more prominent toe drag marks, while the PIN and PNSC groups had fewer drag marks with occasional toe impressions. Behavioral aligned with histological findings, highlighting the superior efficacy of the PINN group in motor function recovery. Furthermore, electrophysiological recordings demonstrated that the PINN group exhibited shorter latencies and higher amplitudes, indicating that implantation of the neuroactive network tissue effectively promoted the formation of relay neurons and the conduction of electrical signals (Fig. 6E).

### 2.6 Single-cell RNA seq unveils the role of neuroactive network tissue transplantation in reconstructing the early local microenvironment and modulating immune response following SCI

**Fig. 8:**
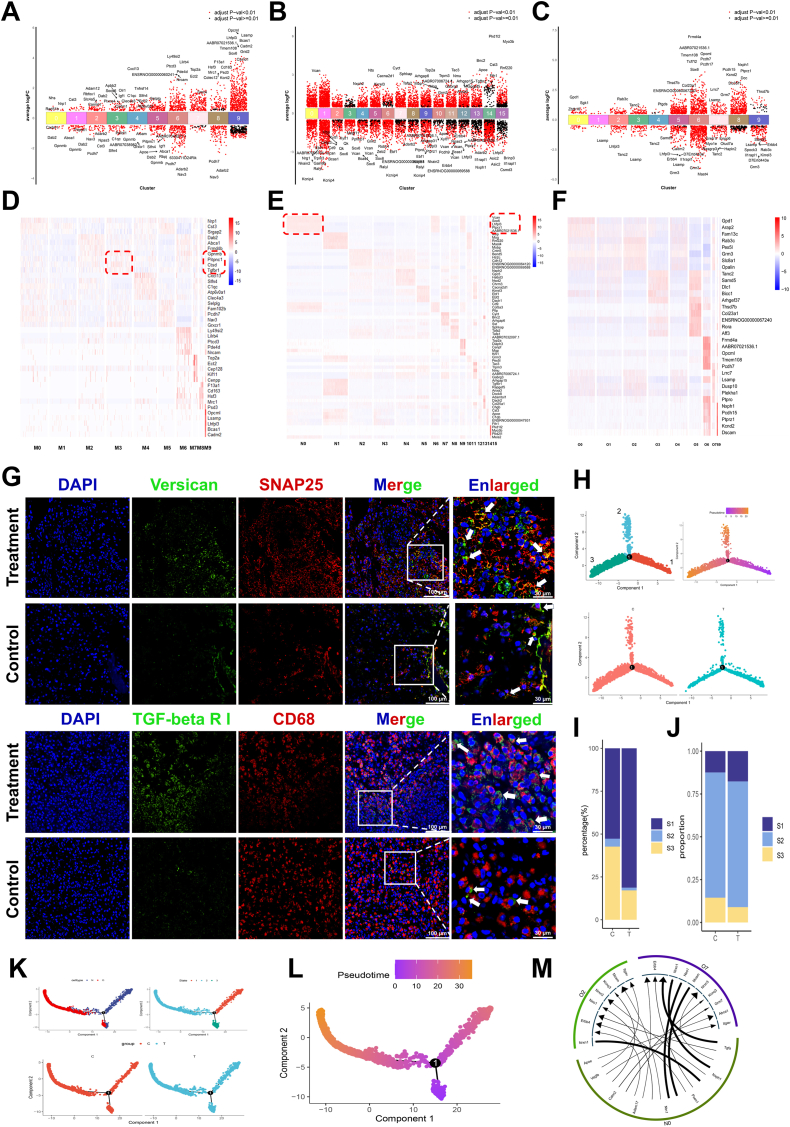
Effects of neural active network transplantation on the development, phenotype, and function of microglia/macrophage, neuron, and oligodendrocyte subtypes in the injured area. (A, B, C) DEGs in each microglia/macrophage, neuron and oligodendrocyte subtype. (D, E, F) Heatmap of FEGs in each microglia/macrophage, neuron and oligodendrocyte subtype. the top 5 genes in each cluster are presented, along with their relative expression levels across all subtypes. (G) Immunofluorescence staining of neuron subcluster (N0) and microglia/macrophage subcluster (M3) in the injured spinal cord tissue of different groups. Scale bars, 100 μm, 30 μm (H) Top-left plot shows the trajectory of microglia/macrophage predicted by Monocle2, colored by their states. Top-right plot shows the trajectory of microglia/macrophage predicted by monocle2 colored by pseudotime. The below plot shows differentiation trajectories of negative control and treatment groups. (I–J) Percentage of microglia/macrophage, neuron, and oligodendrocyte in negative control and treatment groups in each state. (K) The differentiation trajectory of neurons and oligodendrocytes. Red dots represent oligodendrocytes, and blue dots represent neurons. The differentiation trajectories of neurons and oligodendrocytes in the negative control and treatment groups. (L) The trajectory of neurons and oligodendrocytes predicted by monocle2 colored by pseudotime. (M) Single-cell transcriptomic analysis revealed intercellular communication networks between key neuronal and oligodendrocyte subpopulations. (For interpretation of the references to color in this figure legend, the reader is referred to the Web version of this article.)

To further explore the protective effects of neuroactive network tissue transplantation on injured cells and its immunoregulatory mechanisms through the establishment of a local regenerative microenvironment during the early stages of SCI, as well as to uncover cellular reprogramming and functional heterogeneity within the injured area, we conducted single-cell sequencing analysis of spinal cord tissues from the Control (C) and PINN (T) groups at day 7 post-injury. Using the Seurat algorithm combining with SingleR and cell-specific markers, we clustered and annotated these cells within the spinal cord tissue. The results revealed that the tissue microenvironment was primarily composed of seven cell types: neurons, astrocytes, oligodendrocytes, microglia/macrophages, fibroblasts, and NK cells (Fig. 7A–B and Fig. S9A) Comparative analysis of cell proportions between the PINN and Control groups revealed a notable reduction in microglia/macrophages in the PINN group, accompanied by a substantial increase in neurons and oligodendrocytes (Fig. 8C and D). These findings indicated that neuroactive network transplantation reduces the early recruitment and activation of inflammatory cells, suppresses the inflammatory response, and promotes the survival of neurons and oligodendrocytes. Furthermore, ssGSEA analysis revealed a significant reduction in the activation of oxidative stress-related genes in neurons and oligodendrocytes following treatment (Fig. S9B–C).

#### 2.6.1 Microglia/macrophage heterogeneity in spinal cord tissue following neuroactive network tissue transplantation

To further examine alterations in microglia and macrophages following neuroactive network transplantation in spinal cord tissue, we first conducted subtype identification using the SNN algorithm. This analysis identified 10 distinct microglia/macrophage subtypes (Fig. 8E). Comparison of cell proportions between the negative control and PINN groups revealed that microglia/macrophage subtypes M0, M2, M4, M6, M7, M8, and M9 were predominantly enriched in the negative control group, whereas M3 and M5 were significantly elevated in the PINN group (Fig. 8F–G). These findings suggest that the emergence of M3 and M5 microglia/macrophage subtypes may be closely associated with neuroactive network tissue transplantation. After identifying the FEGs and DEGs for each subtype, we found that these genes showed significant independence, with some genes being shared across different subtypes, indicating gene expression differences among subtypes (Fig. 8A and D). GO enrichment analysis further indicated that macrophages in the negative control group were primarily involved in processes related to cell motility, phagocytosis, and nervous system development, whereas those in the PINN group were predominantly associated with bone tissue development, lymphocyte proliferation, and coagulation functions (Fig. S10A–B). These findings suggest that microglia/macrophage subtypes in the PINN group possess a unique potential for bone repair. To further explore the differentiation patterns of macrophages, we used the Monocle2 algorithm to track their differentiation trajectories. The results showed that microglia/macrophages primarily exist in three distinct states: S1, S2, and S3(Fig. 8H and Fig. S13A). Compared to the negative control group, microglia/macrophages in the PINN group were predominantly concentrated in state S1, where genes highly expressed in this state were closely associated with immune cell activation, chemotaxis, and neuronal repair (Fig. 8I and Fig. S10C–D). These findings suggest that neuroactive network tissue transplantation can effectively remodel the local immune microenvironment in the spinal cord, promoting the differentiation of microglia/macrophages into reparative phenotypes that support tissue regeneration.

**Fig. 9:**
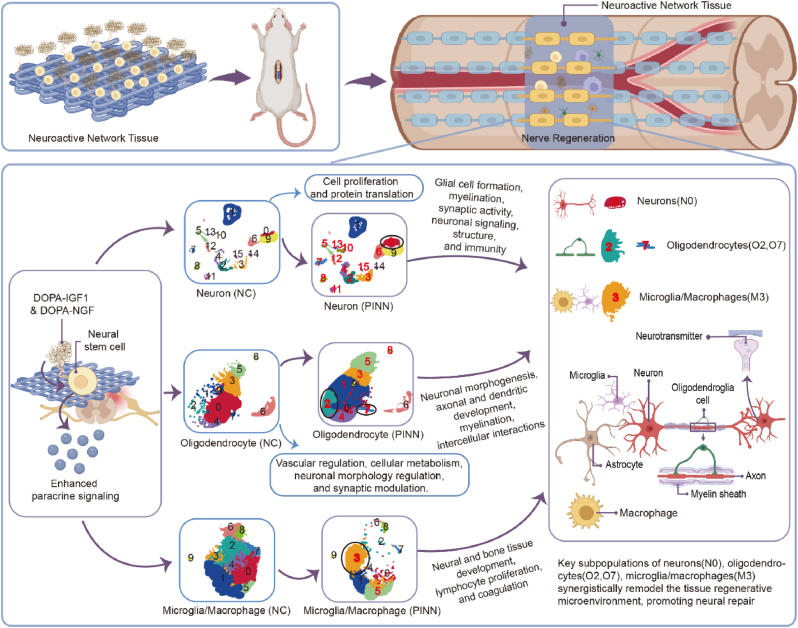
Key subpopulations of neurons(N0), oligodendrocytes (O2, O7), microglia/macrophages(M3) synergistically remodel the tissue regenerative microenvironment, promoting neural repair.

In the PINN group, the M3 subtype of microglia/macrophages exhibited the highest proportion of cell numbers, and therefore, we selected the subtype M3 as a representative for subsequent studies. To investigate the intrinsic gene regulatory mechanisms of the M3 subtype, we applied the CSN algorithm to construct its gene regulatory network and analyzed the nodal degrees within the network. The top 10 driver genes identified in the M3 subtype predominantly included secretory proteins (e.g., IGF1, C1qb, Cst3), transcription factors (e.g., Nipbl, Ash1l, Mef2c), and membrane surface proteins (e.g., Tbxas1, Ptprj, Csf1r) (Fig. S15). Furthermore, we utilized the DeepWalk and GMIGAGO algorithms to identify functional modules within the network. Our analysis revealed that the M3 subpopulation plays a pivotal role in tissue repair, cellular development and metabolism, intracellular and extracellular signaling, gene regulation, and immune response (Fig. S14A–E). These results suggest that after neuroactive network tissue transplantation, microglia/macrophage’ subtype M3 may contribute to local spinal cord tissue repair and immune regulation. Furthermore, differentiation trajectory analysis revealed that M3 macrophages predominantly exist in the state S1, with genes highly expressed in this state associated with immune cell activation, chemotaxis, and neuronal repair. This further supports the critical role of microglia/macrophage’ subtype M3 in tissue repair (Fig. S10E). Immunofluorescence staining further confirmed the presence of the M3 key subpopulation at the histological level. Following transplantation of the neuroactive network tissue, local inflammatory responses were attenuated, accompanied by an increase in the abundance of the M3 subpopulation (Fig. 9G).

#### 2.6.2 Neural system Reorganization in spinal cord tissue after neuroactive network tissue transplantation

We performed subtype identification of neurons and oligodendrocytes, identifying 16 subtypes of neurons and 10 subtypes of oligodendrocytes (Fig. 8H and J). Cell proportions analysis in the negative control and PINN groups revealed that oligodendrocyte subtypes O0, O6, and O9 were predominantly present in the negative control group, while subtypes O1, O2, O3, O4, O5, O7, and O8 were more abundant in the PINN group. For neurons, subtypes N9 and N14 were predominantly found in the negative control group, whereas subtypes N0, N2, N3, N4, N5, N6, N7, N8, N10, N11, N12, N13, and N15 were primarily present in the PINN group (Fig. 8I and 8K-M). These results suggest that neuroactive network tissue transplantation significantly enhances tissue regeneration and repair by promoting the involvement of neurons and oligodendrocytes. DEGs and FEGs revealed substantial differences between neuronal and oligodendrocyte subtypes, with each subpopulation exhibiting distinct expression profiles (Fig. 9B–C and 9E-F). GO enrichment analysis revealed that neuronal subtypes in the negative control group were primarily involved in cell proliferation and protein translation, while those in the PINN group were predominantly associated with functions such as glial cell and myelin formation, synaptic signaling, neuronal signaling, the development and regulation of neuronal morphology and structure, and immune regulation (Fig. S11A–B). The oligodendrocyte subtypes in the negative control group were primarily involved in vascular regulation, cellular metabolism, neuronal morphology regulation, and synaptic modulation. In contrast, the subtypes in the PINN group were mainly associated with processes such as neuronal morphogenesis, axon and dendrite development, myelination, intercellular interactions, and ion regulation (Fig. S11C–D). These findings suggest that, following neuroactive network tissue transplantation, oligodendrocytes and neurons in the spinal cord may cooperate to enhance myelination, thereby contributing to the reconstruction of neural circuits. Given the shared origin of oligodendrocytes and neurons from common NSCs, we constructed a differentiation trajectory model that incorporates both cell types to more accurately simulate the developmental processes of the central nervous system. Differentiation trajectory analysis identified a common starting point (S1) for both oligodendrocytes and neurons, with each differentiating into distinct lineages. Compared to the negative control group, the proportion of cells in the state S1 was significantly higher in the PINN group, with genes highly expressed in this low-differentiation state S1 primarily associated with neurogenesis and synaptic signaling (Fig. 9J–L and Fig. S13B). This suggests that, following neural activity network transplantation, the number of stem-like cells in the spinal cord increases significantly, likely derived from the NSCs population that we transplanted and recruited. Additionally, functional enrichment analysis of the three cell states further supports this hypothesis and aligns with previous experimental findings (Fig. S12A–B).

Following neuroactive network tissue transplantation, neurons in the spinal cord show increased activation and contribute to myelin regeneration. To investigate the mechanisms underlying neuronal myelin repair post-transplantation, we focused on the N0 subpopulation, which is primarily involved in neurogenesis and myelination. Immunofluorescence staining confirmed the presence of the N0 subpopulation. Following transplantation of the neuroactive network tissue, neuronal survival was enhanced, accompanied by an increased proportion of this subpopulation (Fig. 9G). Additionally, because oligodendrocytes play a crucial synergistic role with neurons in central nervous system regeneration, we also conducted an in-depth analysis of the two oligodendrocyte subtypes, O2 and O7, which exhibited the highest cell proportions after transplantation. We constructed gene regulatory networks for the N0, O2, and O7 subtypes and analyzed the degree of network nodes. The top 10 driving genes in the subtype N0, based on degree analysis, include membrane proteins (e.g., Lsamp, Lhfpl3, Csmd3), transcription factors (e.g., Myt1, Zeb1, Sox5), and secretory proteins (e.g., Ptprz1, Xylt1, Vcan). Notably, these genes differ from those driving the oligodendrocyte subtypes. Subtypes O2 and O7 share similar membrane proteins, but their secretory proteins and transcription factors are relatively distinct (Fig. S15). Further analysis of network functional modules revealed that all three cell subpopulations are involved in processes such as RNA binding, neurodevelopment, cellular morphological changes, and signal transduction, although their activation levels differ. During neurodevelopment, Subtype N0 is primarily involved in processes related to neuronal development, whereas Subtypes O2 and O7 are more focused on myelination and assembly (Fig. S14F–J). Cellular communication between neuronal subtype N0 and oligodendrocyte subtypes O2 and O7 is depicted, highlighting dynamic cell–cell interactions during the spinal cord repair process(Fig. 9M). These findings further confirm that neuroactive network tissue transplantation supports the formation and survival of neurons and oligodendrocytes at the site of SCI, and their synergistic interactions facilitate neural circuit remodeling and tissue repair.

## 3 Discussion

SCI causes irreversible sensory and motor deficits, with effective treatment remaining challenging due to extensive neuronal loss, disrupted neural circuits, and a complex inhibitory microenvironment that hinders tissue regeneration b), [18]. To address these challenges, this study developed a neuroactive network tissue by integrating NSCs with an oriented nanofiber scaffold functionalized with a dual-factor neuroregenerative bioactive coating. This tissue was then transplanted to the injury site to facilitate spinal cord repair. By harnessing the enhanced paracrine effects of NSCs alongside a neuroregenerative bioactive coating, this approach first optimized the local microenvironment, reducing oxidative stress, inflammation and programmed neuronal death. In subsequent stages, it promoted neurogenesis, vascular regeneration, and axonal growth, facilitating integration with surrounding healthy tissues and supporting neural circuit reconstruction. By sequentially and multidimensionally optimizing the regenerative microenvironment, this approach significantly enhanced SCI repair outcomes.

Electrospinning technology, widely used in neural tissue engineering, can promote axonal alignment and regulate cellular behavior through its specialized topographical structures [19]. PGCL-based oriented nanofiber scaffolds mimicking the extracellular matrix promoted axonal growth and neural circuit reconstruction. These scaffolds further demonstrated excellent biocompatibility and cellular support. To overcome the limitations of soluble growth factors, this study used mussel-inspired motifs to engineer DOPA-IGF1 and DOPA-NGF, introducing the innovative concept of growth factor density [10b]. By optimizing the modification concentration, growth factor coatings were immobilized on nanofiber scaffolds and combined with NSCs to create neuroactive network tissue with neuroinductive and tissue-regenerative functions. This approach enhanced the biological effects of growth factors while reducing costs [20]. Compared to soluble growth factors, immobilized ones show stronger effects and better efficacy due to localized high concentrations, enhanced receptor dimerization, and reduced receptor downregulation d), [21]. Experimental results showed that the dual-factor neuroregenerative bioactive coating significantly enhanced the paracrine effects of NSCs, effectively promoting new neurons formation, and improving SCI repair outcomes.

After SCI, microglial cells are rapidly activated, while macrophages infiltrate the injury site due to blood-spinal cord barrier disruption, peaking around seven days post-injury [22]. The inflammatory cytokines released by these immune cells, along with ischemia and oxidative stress, create an inhibitory microenvironment that induces programmed cell death and impedes tissue regeneration [23]. Modulating the early inhibitory microenvironment is critical for effective tissue repair. Building on the neuroregenerative potential and paracrine functions of NSCs, this study developed a neuroactive network tissue. With sustained stimulation from a dual-factor neuroregenerative bioactive coating, the system significantly enhanced the paracrine effects of NSCs, remodeled the extracellular matrix, and promoted neuronal differentiation and myelin regeneration. In vitro, the neuroactive network tissue reduced ROS production, preserved mitochondrial function, and enhanced antioxidant capacity. It improved the immune microenvironment by modulating macrophage polarization, decreased programmed cell death, and significantly promoted neuronal generation and axonal extension. In vivo, transplantation led to spinal cord tissue recovery, increased neurogenesis, axonal and vascular regeneration, reduced scar formation, and attenuated inflammatory responses. These findings confirm the efficacy of multidimensional regenerative microenvironment construction in tissue repair and neural circuits integration.

RNA-Seq revealed the mechanisms through which neuroactive network tissue regulates NSC behavior. Our findings provide strong evidence for the role of bifactorial bioactive coatings in modulating neurogenesis and tissue repair. Specifically, the dual-factor neuroregenerative bioactive coating upregulated the expression of these genes involved in neurogenesis, synaptogenesis, antioxidant stress response, and ROS metabolism. Moreover, the coating enhanced the secretion of paracrine molecules related to angiogenesis and tissue repair while inhibiting the pro-inflammatory factors. Compared to the negative control group, DEGs were primarily enriched in key regions such as the extracellular matrix, extracellular vesicles, and neuronal axons, and were involved in processes like neurogenesis, angiogenesis, and axon growth guidance. Further GSEA Analysis showed upregulation of gene sets related to axon growth and extracellular matrix-receptor interactions. KEGG pathway analysis indicated that the effect is likely mediated through the PI3K-Akt-mTOR signaling pathway. These findings highlight he therapeutic potential of neuroactive network tissue in enhancing NSC paracrine effects, modulating the immune microenvironment, and promoting neurogenesis, axonal growth, and tissue regeneration.

A key challenge in SCI repair is the complex inhibitory microenvironment, involving dynamic interactions among multiple cell types, which our therapeutic approach targets b), a), [24]. While most studies focus on histological and behavioral recovery, some have adopted multi-omics approaches to better understand the therapeutic effects post-injury [8b,9b]. Using scRNA seq, we analyzed how neuroactive network tissue exerts early protective effects on local cells and modulates immune responses post-injury, revealing cellular reprogramming as well as functional and phenotypic heterogeneity at the injury site. The results show a decrease in microglia and macrophages proportions in the treatment group, with an increase in neurons and oligodendrocytes. Additionally, the activation of oxidative stress-related genes in neurons and oligodendrocytes was significantly reduced, indicating that the treatment mitigated inflammation and enhanced cell survival. Subtypes analysis of microglia/macrophage, neurons, and microglia subtypes revealed that microglia/macrophage subtypes in the treatment group displayed functional phenotypes linked to neural and bone tissue development, lymphocyte proliferation, and coagulation. Trajectory analysis showed that microglia/macrophages in the PINN group were primarily concentrated in the state S1, with genes highly expressed in the state S1 closely related to immune cell activation and neuronal repair. This finding suggests that neural activity network transplantation reshapes the local immune microenvironment and promotes the microglia/macrophages differentiation toward a tissue-repair phenotype. The synergy between neurons and oligodendrocytes is crucial for central nervous system regeneration and neural circuit reconstruction. Differentiation trajectories analysis revealed that oligodendrocytes and neurons share a common origin at the state S1, with a significant increase in cell state S1 proportions in the treatment group. This suggests that neural activity network transplantation remodels the spinal cord tissue microenvironment, allowing both transplanted and endogenously NSCs to differentiate into neurons and oligodendrocytes. These cells then synergize to promote tissue repair at the injury site. These findings offer new target cells and therapeutic strategies to enhance post-transplantation outcomes for SCI (Fig. 9).

## 4 Conclusion

This study employed a combinatorial therapeutic strategy to create neuroactive network tissue by integrating NSCs with an oriented nanofiber scaffold functionalized with a dual-factor bioactive coating. In the early phase, the dual-factor neuroregenerative bioactive coating enhanced NSCs paracrine effects, reducing oxidative stress, inflammation and inhibiting neuronal apoptosis. Later, it promoted neurogenesis, axonal growth, and neural circuit reconstruction. ScRNA seq analysis further showed improved the local microenvironment and promoted tissue regeneration by attenuating immune cell activation and enhancing the survival of neurons and oligodendrocytes. Both in vitro and in vivo experiments confirmed that the neuroactive network tissue remodeled the immune microenvironment at the SCI site, promoted cellular differentiation and repair, and provided a multidimensional regenerative strategy. Our study presents a novel therapeutic approach for SCI, demonstrating the significant potential of neuroactive network tissue in enhancing regeneration.

## 5 Experimental section

### 5.1 Preparation of immobilized recombinant growth factors

Building upon our previous work and existing literature, we have developed an improved protocol for the preparation of immobilized recombinant growth factors. Using genetic engineering techniques, a small pentapeptide tag containing tyrosine residues (Tyr-Lys-Tyr-Lys-Tyr, YKYKY) was introduced at the C-terminus of the IGF-1 and NGF sequences. These modified sequences were then recombined with the pET-15b vector to construct expression plasmids (Sangon Biotech, China), followed by prokaryotic expression in *Escherichia coli*. Colonies containing the recombinant plasmids were inoculated into LB medium supplemented with 100 μg mL^−1^ ampicillin and incubated overnight at 37 °C with shaking. Subsequently, the cultures were transferred to 1 L flasks containing YTA medium (also supplemented with 100 μg mL^−1^ ampicillin) and further incubated at 37 °C with shaking. When the optical density at 600 nm (OD600) reached 0.6–0.8, protein expression was induced by the addition of isopropyl β-D-thiogalactopyranoside (IPTG) to a final concentration of 0.6 mM, followed by overnight incubation. After ultrasonic crushing, the resulting inclusion bodies were harvested by centrifugation, washed with 1.5 M guanidine hydrochloride (Gdn-HCl), and dissolved in 6 M Gdn-HCl at 4 °C overnight. The dissolved inclusion body proteins were subjected to refolding, concentration, and purification using a Ni-NTA affinity chromatography column. Further purification was performed via sieve chromatography (Nanomicro, China). The purified proteins were aliquoted into 1.5 mL Eppendorf tubes. Finally, the protein solutions were lyophilized into powder using a freeze dryer and stored at −80 °C for subsequent use.

### 5.2 Preparation of neuroregenerative bioactive coating

We defined the amount of recombinant growth factor per unit area as the growth factor density. For example, at a growth factor density of 100 ng cm^−2^, the lyophilized immobilized recombinant growth factor powder was diluted in PBS to a concentration of 1 μg mL^−1^. Using a 24-well plate (with an approximate well area of 2 cm^2^), the following components were added to each well: recombinant growth factor (1 μg mL^−1^, 200 μL), tyrosine hydroxylase (10 UI μL^−1^, 1 μL) (Sigma–Aldrich, USA), ascorbic acid (5 mg mL^−1^, 300 μL) (Solarbio, China), and PBS (499 μL). The mixture was thoroughly homogenized, and the pH of the solution was adjusted to 7.2 using 1 mol L^−1^ NaOH. The plate was then left at room temperature for 2 h, followed by further adjustment of the pH to 8.5. The mixture was incubated at 4 °C overnight.

### 5.3 SDS-PAGE and western blot

The immobilized recombinant growth factor was analyzed at various stages of preparation using SDS-PAGE. Samples included: pre-induction, post-induction, supernatant after centrifugation, pellet after centrifugation, refolded protein solution, flow-through liquid, wash liquid, and elution liquid. Protein bands were visualized using Coomassie Brilliant Blue staining, followed by destaining with ethanol at 60 °C. For Western blot analysis, protein bands separated by SDS-PAGE were transferred onto a polyvinylidene difluoride (PVDF) membrane (Roche Tech, Switzerland) using an electroblotting apparatus (Bio-Rad, USA) in Tris-glycine buffer at a constant current of 200 mA for 40 min. The membrane was then blocked with TBST containing 10 % fetal bovine serum (FBS) for 1 h at room temperature. The membrane was incubated with the primary antibody overnight at 4 °C. Following TBST washing, the membrane was incubated with the secondary antibody for 1 h, washed again with TBST, and subsequently visualized using an enhanced chemiluminescence (ECL) detection system.

### 5.4 Preparation of PGCL polymer material interface

The PGCL polymer material (GA:CL = 10:90, GA:CL = 20:80, GA:CL = 30:70, Mn = 150,000) was provided by the Regenerative Medicine Materials Research Group at the Changchun Institute of Applied Chemistry, Chinese Academy of Sciences. To prepare a 3 % (w/v) solution, PGCL was dissolved in hexafluoroisopropanol (HFIP). The solution was evenly applied onto glass slides to form a polymer interface with a thickness of 0.1 mm. The coated slides were then vacuum-dried at room temperature for 48 h.

### 5.5 Adhesion and stability of immobilized recombinant growth factors

The adhesion and stability of recombinant growth factors were assessed by using double antibody sandwich ELISA (Abcam, ab108873, ab99986, USA). For hydroxylation, a 1 mL reaction system was prepared, and the hydroxylated recombinant growth factor solutions from different concentration groups were transferred into Eppendorf tubes. Each well was washed three times with 1 mL PBS, and the wash solutions were collected. The collected liquids from each group were added to the corresponding assay wells, and the residual growth factor concentrations in the supernatants were measured. Adhesion amount and adhesion efficiency were calculated based on the initial application amount of growth factors in each group. For stability assessment, the hydroxylated samples were placed into 24-well plates according to different groups, with 1 mL PBS added to each well. At specified time intervals, the supernatants were collected for growth factor content determination, and fresh PBS was replenished. The cumulative release of proteins was calculated over time to evaluate the stability of the growth factors. All samples were diluted to appropriate concentrations for detection.

### 5.6 Isolation, culture, and identification of NSCs

Following a modified protocol based on previous literature, NSCs were isolated from the hippocampus of embryonic day 13–15 SPF-grade Sprague-Dawley rat fetuses bought from Vital River Company (Beijing, China). The hippocampus was carefully dissected to remove impurities and microvessels. The brain tissue was finely minced and triturated 20 times using a Pasteur pipette, followed by 10 triturations with a 1 mL pipette tip, with all steps performed on ice. The tissue suspension was filtered sequentially through 70 μm and 40 μm cell strainers, and the filtrate was centrifuged at 1500 rpm for 5 min at room temperature. The resulting cells were resuspended in NSC proliferation medium containing DMEM/F12 (Gibco, A4192001, USA), 1 % penicillin-streptomycin (PS, Sigma-Aldrich, USA), 1 % glutamine (Gibco, USA), 20 ng mL^−1^ basic fibroblast growth factor (bFGF, Solarbio, China), and 20 ng mL^−1^ epidermal growth factor (EGF, Solarbio, China). Cells were cultured at 37 °C in a 5 % CO_2_ incubator. Cell cultures were observed daily under an optical microscope, and images were recorded. When the diameter of neurospheres reached approximately 200 μm and the center appeared dark, passaging was performed. Neurospheres were dissociated using 2 mL Accutase enzyme (Gibco, A1110501, USA), and the dissociated cells were further cultured. Third to fifth-generation NSCs in good condition were selected for subsequent experiments. NSCs were identified with nestin antibody(Abcam, ab92391, USA) as the primary antibody. Cells were further labeled with a secondary antibody(Abcam, ab150078, USA) and DAPI(Servicebio, G1012, China) to confirm NSC identity and visualize cellular morphology.

### 5.7 Cell proliferation

Extracts from different material groups were collected and serially diluted. NSCs were seeded in 96-well plates and cultured for 24 h at 37 °C in a 5 % CO_2_ incubator. After removing the medium, the extracts and their serial dilutions were added to the wells, and the cells were further incubated for another 24 h. Cell proliferation was then assessed using the CCK-8 kit (Abcam, ab228554, USA) and a multifunctional microplate reader to evaluate the biocompatibility of the material. Additionally, two types of immobilized growth factors were applied at varying densities to the bottom of the wells. NSCs were seeded at a uniform density into the wells and cultured. Proliferation was measured on days 1, 3, and 5 using the CCK-8 kit and a multifunctional microplate reader to evaluate the effects of the immobilized growth factors on cell proliferation.

### 5.8 Immunocytochemistry

To investigate the regulatory effects of different growth factor coating densities on NSC differentiation, NSCs were cultured on growth factor-coated surfaces for 7 days. The cells were then processed as follows: Fixed with 4 % paraformaldehyde for 30 min. Permeabilized with 0.5 % Triton X-100 solution (Abcam, ab286840, USA) at room temperature for 30 min. Blocked with 10 % goat serum for 1 h at room temperature. Each step was followed by three PBS washes. After blocking, cells were incubated with the primary antibody Tuj-1(Abcam, ab215037, USA) at 4 °C overnight. On the following day, cells were incubated with a secondary antibody (Abcam, ab150077, USA) and DAPI in the dark at room temperature for 2 h. Finally, samples were visualized and images captured using a laser confocal microscope (Nikon, Japan).

### 5.9 Preparation of oriented electrospun nanofiber network scaffolds

Three different compositions of PGCL polymer materials were dissolved in 3 mL of HFIP to prepare electrospinning solutions with concentrations of 13 wt %, 15 wt %, and 17 wt %. The solutions were loaded into 2.5 mL syringes equipped with 23G needles (outer diameter ∼0.6 mm) and secured in a custom-designed electrospinning apparatus developed by our research group. The high-voltage electrostatic generator was connected to the needle and the rotating collector to create an electric field. Electrospun fibers were collected on aluminum foil substrates. Based on previous research from our group, the spinning distance was set at 13 cm, 15 cm, and 18 cm. Using an orthogonal experimental design, 27 sample groups were fabricated for further analysis.

### 5.10 Characterization of oriented electrospun nanofiber network scaffolds

#### 5.10.1 Morphological analysis with scanning electron microscopy (SEM)

The prepared dry electrospun scaffolds were cut into 1 cm^2^ samples and mounted onto SEM sample holders. After gold sputtering, the samples were observed under SEM (xl30 ESEMFEG, Netherland) at an accelerating voltage of 10 kV. Images were collected from different regions of each sample, and the diameters of 100 individual fibers per sample were measured and analyzed using ImageJ software.

#### 5.10.2 Contact angle measurement

The contact angle of the electrospun scaffold surface was measured using a contact angle measurement system (DSA 10, Germany). A 30 μL droplet of deionized water was gently dispensed onto the scaffold surface, and the angle between the droplet and the scaffold surface was recorded. Changes in the contact angle were observed, and the test was repeated five times at different positions on each sample. Results were averaged to provide statistical analysis.

#### 5.10.3 Mechanical property testing

Mechanical properties of the electrospun scaffolds were tested using an electronic universal testing machine (Instron 1121, USA) at room temperature. Samples were prepared as rectangular specimens with dimensions of 10 mm × 30 mm and placed within a 500-N load cell. The tensile speed was set at 10 mm min^−1^, and the force-displacement data were recorded. Stress-strain curves were generated from the data, and the elastic modulus and tensile strength were calculated.

### 5.11 Characterization of functionalized surface coating of the electrospun scaffolds

As described previously, electrospun scaffolds were surface-functionalized using immobilized recombinant growth factors. X-ray photoelectron spectroscopy (XPS, Thermo, USA) analyzed the surface chemical elements and bonding states to confirm the successful incorporation of growth factors. FT-IR (Bruker Vertex 70, Germany) detected changes in functional groups, verifying the presence of characteristic peaks indicative of successful scaffold modification.

### 5.12 Construction of neuroactive network tissue and characterization of neural induction

Following the previously described method, the electrospun nanofiber network scaffolds were surface-functionalized using the optimal growth factor density to create dual-factor (**D-I&N**), single-factor (**D-NGF, D-IGF1**), and uncoated control (**Contro**l) scaffolds. Each scaffold group was soaked in NSC culture medium for 2 h. NSCs in good condition were then seeded onto the scaffolds at a density of 5 × 10^5^ cells and cultured in low-adhesion cell culture plates to allow cell attachment, growth, and differentiation. This process facilitated the integration of cells into the scaffolds to form neuroactive network tissue. After 7 days and 14 days of cultivation, neural induction effects and axonal directional growth abilities were evaluated using immunofluorescence staining as previously described.

### 5.13 Cellular protective effects of neuroactive network tissue

To investigate the protective effects of neuroactive network tissue via paracrine function in a simulated SCI microenvironment, a series of experiments were conducted using Transwell plates with the following groups: **@NSC + D-N&I**, **@NSC**, **H_2_O_2_+D-N&I**, **H_2_O**_**2**,_ and **Control**. **@NSC + D-N&I** and **@NSC** groups: Neuroactive network tissue functionalized with dual-factor coatings were placed in the upper chambers of the Transwell plates. **H_2_O_2_+D-N&I**, **H_2_O_2_**, and **Control** groups: No samples were placed in the upper chambers. For **@NSC + D-N&I** and **H_2_O_2_+D-N&I** groups, the lower chambers were pre-coated with dual-factor functionalized layers to simulate direct contact effects of the neuroactive coatings on cells. HT22 cells were seeded into the lower chambers of the Transwell plates and incubated for 6h. After this initial incubation, the medium was replaced with 500 μM H_2_O_2_-containing medium in all groups except the **Control**, and the cells were further incubated. Cell viability and stress responses were assessed using Live/Dead staining (cell survival) (Sigma, 17783, USA), DCFH-DA staining (ROS levels) (Beyotime, S0035S, China), and JC-1 fluorescence (mitochondrial membrane potential) (Solarbio, M8650, China).

### 5.14 Immunomodulatory function of neuroactive network tissue

As previously described, macrophages were seeded into the lower chambers of each Transwell group. After 6 h of incubation, the medium was replaced with 100 ng mL^−1^ LPS-containing medium, followed by an additional 48 h of incubation. Cell morphology was observed using fluorescence microscopy, and iNOS expression was evaluated via immunofluorescence staining (Abcam, ab178945, USA).

### 5.15 Animal experiment

All animal experiments were conducted in compliance with the Chinese Ministry of Public Health Guide for Animal Ethics and Welfare. This study was approved by the Animal Ethics Committee of the First Hospital of Jilin University (Ethics No. JDYY20240304).

Female SD rats (350–400 g) were used to establish a spinal cord transection injury model under general anesthesia (RWD, China). Anesthesia was induced with isoflurane (3.5 %, 1 L min^−1^) for 2 min and maintained at 2 % isoflurane with a flow rate of 0.6 L min^−1^. After anesthesia onset, the dorsal spinal column was shaved, disinfected, and a midline incision was made. Subcutaneous tissue and muscles were separated to expose the lamina, which was removed. A 3 mm section of the spinal cord at the T9 segment was carefully excised. Treatments were administered as follows: **PINN group:** Neuroactive network tissue. **PNSC group:** Scaffolds loaded with NSCs. **PIN group:** Scaffolds with dual-factor coating. **PGCL group:** Plain scaffolds. **Control group:** No treatment. The incision was sutured layer by layer, and rats were monitored until recovery from anesthesia. Postoperative care was provided for 8 weeks, including assisting with bladder voiding twice daily until spontaneous urination resumed and administering intramuscular penicillin for 5 consecutive days.

### 5.16 Behavioral assessments

Hind limb motor function recovery in rats was assessed using the BBB (Basso Beattie Bresnahan) locomotor scale and footprint analysis. Two independent observers, blinded to the experimental groupings, evaluated joint movement range, paw fine motor skills, and gait coordination. Assessments began on postoperative day 1 and were performed weekly on the first day of each week for 8 weeks. At the end of the 8-week period, the hind paws of the rats were coated with ink, and the rats were allowed to walk through a narrow corridor lined with white paper. The gait patterns were observed as the rats moved toward a dark box at the end of the corridor. Once the rats entered the box, the white paper was collected, air-dried, and analyzed to record the gait trajectories.

### 5.17 Electrophysiological assessment

Motor evoked potentials (MEPs) were measured using an electromyography and evoked potential recording system. All animals underwent identical anesthesia procedures prior to assessment. For MEP stimulation, bipolar needle electrodes were positioned on the skull surface. Recording needle electrodes were inserted into the tibialis anterior muscles of both hindlimbs, and a subcutaneous needle electrode placed on the back served as the ground. The latency (ms) and peak amplitude (mV) of MEP responses were recorded for each animal and subjected to statistical comparison among groups.

### 5.18 Histology and immunohistochemistry

Eight weeks after SCI, rats were deeply anesthetized with sodium pentobarbital. Cardiac perfusion was performed sequentially with physiological saline followed by 4 % paraformaldehyde. A 3 cm segment of spinal cord tissue, centered on the T9 injury site, was excised and fixed in 4 % paraformaldehyde for 24 h. The tissue was then dehydrated sequentially in 20 % and 30 % sucrose solutions. The dehydrated tissue was sectioned using a Leica cryostat (Leica, Germany). Histological recovery and myelin regeneration were evaluated using hematoxylin-eosin (H&E) (Beyotime, C0105S, China) and Luxol Fast Blue (LFB) (Beyotime, C0633S, China) staining, following established protocols [25]. Immunohistochemical analysis was performed to assess the formation of new neurons (Tuj-1, Abcam, ab215037, USA) (GFAP, Abcam, ab254083, USA) (Versican, Bioss, bs-2533R, China) (SNAP25, Bioss, bsm-60238M, China), axonal (NF200, Cell Signaling Technology, 55453s, USA) and vascular regeneration (RECA-1, Abcam, ab9774, USA), as well as inflammatory cell infiltration at the injury site (CD68, Abcam, ab283654, USA) (IBA1, Abcam, ab283319, USA) (CD68, Bioss, bsm-33056M, China) (TGF beta Receptor I, Bioss, bs-0638R, China) [8b].

### 5.19 Biosafety and in vivo degradation assessment of scaffolds

Four weeks after scaffold implantation, the heart, liver, spleen, lungs, and kidneys were harvested from each experimental group. Tissues were fixed in 4 % paraformaldehyde, embedded in paraffin, sectioned at 5 μm, and stained with hematoxylin and eosin (H&E). Histological morphology was examined under a light microscope to identify pathological changes and evaluate scaffold biosafety. For degradation analysis, identical scaffolds were implanted subcutaneously in the dorsal region of rats. At predetermined time points, implantation sites were photographed, and macroscopic changes in scaffold appearance, size, and surface integrity were assessed to characterize in vivo degradation behavior.

### 5.20 Statistical analysis

All statistical measurements were performed using ImageJ, and data were presented as mean ± standard deviation (SD). Statistical analysis was conducted using SPSS software (SPSS 26.0). Prior to analysis, the Kolmogorov-Smirnov test was applied to assess data for homogeneity of variance and normality. One-way analysis of variance (ANOVA) was used to compare group means, followed by Bonferroni post hoc tests for multiple comparisons. Statistical significance was considered at *p* < 0.05. Graphs were generated using Prism software (GraphPad 8.0).

### 5.21 Bulk RNA seq data analysis

To investigate the transcriptional regulatory effects of single and dual growth factors on NSCs, Bulk RNA seq was performed using the Illumina sequencing platform. Transcript quantification was conducted using the featureCounts function from the Rsubread R package, with the Rattus norvegicus reference genome (mRatBN7.2) as the reference. The resulting dataset provided read counts for all genes in each sample. Differentially expressed genes (DEGs) were identified by applying the DESeq2 algorithm, using the read counts obtained in the previous step as input. Comparisons were made between the negative control group and experimental groups, with thresholds set at p < 0.05 and |log2FC| > 0.263. The DESeq2 algorithm was implemented using the DESeq function from the DESeq2 R package [26].

### 5.22 scRNA seq data analysis

#### 5.22.1 Single cell sample preparation

At 1 week after SCI, spinal cord tissues from the **Control** and **PINN** groups were collected for scRNA Seq analysis. This aimed to evaluate the intrinsic mechanisms by which neuroactive network tissue transplantation protects local cells and regulates immune responses through the reconstruction of a regenerative microenvironment at the injury site during the early stages of SCI. Rats were deeply anesthetized, and a 1 cm segment of spinal cord tissue centered on the T9 injury site was carefully excised. The dura mater and surface blood vessels were removed, and the tissue was dried to eliminate excess moisture before being rapidly frozen in liquid nitrogen.

#### 5.22.2 Single cell RNA seq

Protoplast suspensions were prepared and loaded onto Chromium microfluidic chips utilizing the 30 v3 chemistry, with barcoding performed via a 10X Chromium Controller (10X Genomics). Full-length cDNA from the barcoded samples underwent reverse transcription and library construction according to the manufacturer's guidelines for the Chromium 30 v3 kit (10X Genomics). Sequencing was carried out on an Illumina NovaSeq 6000 platform, following standard Illumina protocols.

#### 5.22.3 Single-cell data quantification and data preprocessing

For the sscRNA-seq data analysis, the raw sequencing data generated from the Illumina NovaSeq 6000 platform were first subjected to quality control (QC) and preprocessing steps to ensure the integrity and accuracy of downstream analyses.

1 **Single-Cell Data Quantification**

The raw sequencing data, provided in fastq format, were processed using **Cell Ranger** (version 7.0.0, 10X Genomics). This software aligns the reads to the reference genome, calculates unique molecular identifier (UMI) counts for each gene, and generates a gene expression matrix. The reference genome used for alignment was the **Rattus norvegicus reference genome mRatBN7.2**.

2. **Single-Cell Gene Expression Profile Preprocessing**

The gene expression matrix was processed using the Seurat package (version 4.3.1) in R [27]. Cells were identified as low quality and excluded if they met any of the following criteria.

(1) having fewer than 500 or more than 3500 UMIs;
(2) having fewer than 200 detected genes per cell;
(3) more than 10 % of UMIs originating from mitochondrial genes.

Following the exclusion of low-quality cells, a total of 47725 cells were retained for subsequent analysis. Subsequently, UMI counts were normalized using the "NormalizeData" function from the Seurat package. The 3000 genes with the highest entropy scores were selected as the highly variable feature genes. Highly variable feature genes were identified using the “Get_entropy” function from the IEntropy package [28]. Lastly, the gene expression of highly variable feature genes were scaled and centered using the "ScaleData" function in the Seurat package to ensure proper normalization across the dataset.

#### 5.22.4 Single-cell annotation in the spinal cord tissue microenvironment

Using the scaled data mentioned above, the top 10 principal components were computed to identify the primary sources of variation and reduce noise in the data through the RunPCA function. Cells were grouped using the SNN algorithm with the FindClusters function, where a resolution parameter of 0.7 was applied to optimize the number of clusters and capture the majority of the biological variability. For visualization purposes, dimensionality reduction was performed using the UMAP technique with the RunUMAP function. All three functions mentioned above were implemented in the Seurat R package. Finally, cell type identities for each cluster were assigned using the R package SingleR (release: 2.0.0) with annotations based on the "label.fine" method and the built-in reference dataset MouseRNAseqData, along with known cell markers provided in Supplementary [29].

#### 5.22.5 Microglia/macrophage, Neuron, and Oligodendrocyte subtype identification

The gene expression profiles of **Microglia/Macrophages**, **Neurons**, and **Oligodendrocytes** were preprocessed using the following steps: **Normalization:** Gene expression was standardized using the **NormalizeData** algorithm. **High-Variance Gene Identification:** Highly variable genes were identified with the **IEntropy** algorithm. **Scaling:** Data normalization was completed using the **Scale** algorithm. The parameters matched those from the *Single-Cell Gene Expression Profile Preprocessing* step. Subtypes were identified following the workflow described in the *Single-Cell Annotation in the Spinal Cord Tissue Microenvironment* step, with clustering performed using the **FindClusters** function (resolution = 0.5).

#### 5.22.6 Identification of differentially expressed genes and feature genes

Based on the gene expression data of three cell types, each subtype was treated as an experimental group, with the other subtypes in this cell type serving as the negative control group. DEGs for each subtype were identified using Wilcoxon tests. Specifically, genes with a logFC >0.5 and P < 0.05 were considered up-regulated DEGs, while genes with a logFC < −0.5 and P < 0.05 were categorized as down-regulated DEGs.

ROC curves were employed to identify featured differential expressed genes that demonstrated strong discriminatory ability across subtypes, with a filtering threshold of logFC >0.5 and AUC >0.75.

The Wilcoxon test and AUC value calculations were implemented using Seuret “FindAllMarkers” function.

##### 5.22.6.1 Pseudo-time analysis

Pseudotime analysis of each cell types was performed using the R package Monocle (version 2.28.0). Temporal differential genes (TDGs) were identified as the top 3000 significant genes (q < 0.05) in the analyzed cells, using the differential Gene Test function. Using the identified TDGs, cell differentiation trajectories were constructed in Monocle by applying dimensionality reduction and cell ordering with the default parameters.

##### 5.22.6.2 Regulatory network construction and network module identification

we first isolated Microglia/Macrophage, Neuron, Oligodendrocyte expression data from the scRNA-seq dataset and then constructed a single-cell gene regulatory network for each macrophage subtype using the Cell Specific Network (CSN) algorithm [30].

Specifically, the gene regulatory relationships were computed using the CSN algorithm, with the following formula:

$${\rho_{xy}}_{=\frac{n_{xy}^{\left(k\right)}}{n}-\frac{n_{x}^{\left(k\right)}}{n}\cdot \frac{n_{y}^{\left(k\right)}}{n}}$$

In the equation, x denotes gene x, y represents gene y, k refers to cell k, and n rindicates the total number of cells in the region being analyzed.

Based on the single-cell gene regulatory networks of Microglia/Macrophage subtypes M3, Neuron subtypes N0, Oligodendrocyte O2, O7 genes that were involved in at least one regulatory interaction with other genes in 50 % of the cells of each subtype were designated as driver genes. Additionally, the network of regulatory relationships among these driver genes, present in 50 % of the cells within a specific subtype, was defined as the subtype-specific gene regulatory network (ssGRN).

To uncover the functional gene modules within ssGRNs, embedding vectors for each gene in the ssGRN were generated using the Deepwalk algorithm. We then computed the gene embedding similarities using cosine similarity and constructed a gene similarity adjacency matrix. Subsequently, the GMIGAGO algorithm was applied to identify gene modules [31]. For each identified gene module, GO enrichment analysis was performed, and the top 30 GO terms were selected based on ascending P-values. For each GO term, the highest -log P value across all gene modules was defined as the indicator of its enrichment level within a specific subtype.

#### 5.22.7 Cell–cell communication analysis using the iTalk algorithm

The iTalk algorithm was employed to quantify cell–cell communication between the neuronal subtype N0 and the oligodendrocyte subtypes O2 and O7. Significant ligand–receptor interactions were identified, comprising the top 20 predicted pairs with P < 0.05 and an average FPKM >1.

## CRediT authorship contribution statement

**Tianyi Liu:** Writing – review & editing, Writing – original draft, Investigation, Data curation. **Wenhao Zhu:** Formal analysis, Data curation. **Zheng Wan:** Resources, Project administration. **Cong Fu:** Resources, Methodology. **Xiaoyu Zhang:** Methodology, Investigation. **Wenzhong Li:** Project administration, Investigation. **Wenchen Li:** Resources, Formal analysis. **Zhenxu Wu:** Supervision, Resources, Methodology. **Min Guo:** Software, Resources. **Mengtuan Long:** Resources, Project administration. **Feiyang Yang:** Validation, Data curation. **Hongyu Chen:** Writing – original draft, Resources. **Xingcheng Yi:** Writing – original draft, Software, Methodology, Data curation. **Honglei Wang:** Software, Resources, Project administration. **Peibiao Zhang:** Software, Project administration, Investigation. **Haifeng Wang:** Writing – original draft, Validation, Supervision.

## Declaration of Competing interest

The authors declare no conflict of interest.

## Data Availability

Data will be made available on request.
