# Supplementary material for: Neuroactive network tissue based on dual-factor neuroregenerative bioactive coating scaffolds and neural stem cells for spinal cord injury repair
Source: Mater Today Bio. 2025 Aug 5;34:102172. doi: 10.1016/j.mtbio.2025.102172 (PMC12357159; doi:10.1016/j.mtbio.2025.102172)
Supplement: Multimedia component 1 [file mmc1.docx]

**Neuroactive Network Tissue Based on Dual-Factor Neuroregenerative Bioactive Coating Scaffolds and Neural Stem Cells for Spinal Cord Injury Repair**

*Tianyi Liu, Wenhao Zhu, Zheng Wan, Cong Fu, Xiaoyu Zhang, Wenzhong Li, Wenchen Li, Zhenxu Wu, Min Guo, Mengtuan Long, Feiyang Yang, Hongyu Chen, Xingcheng Yi*, Honglei Wang*, Peibiao Zhang*, Haifeng Wang**


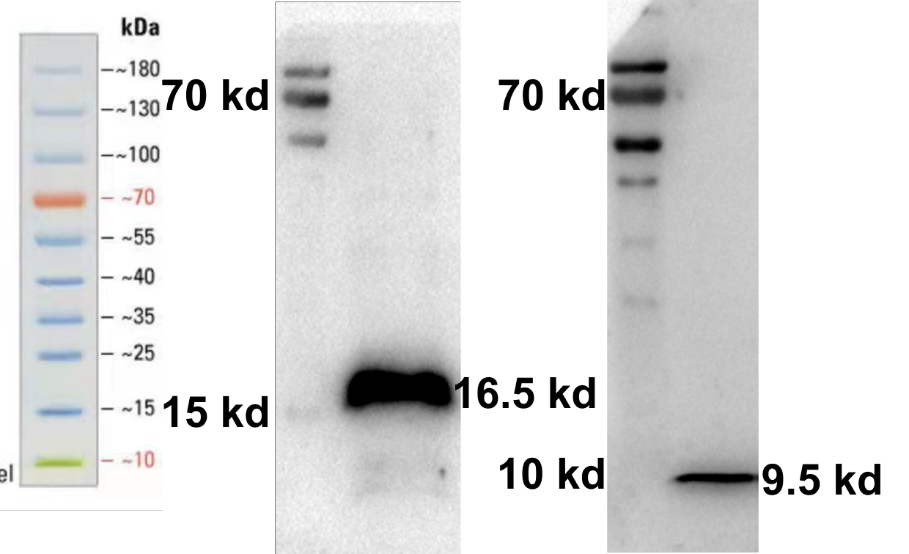


F S1. Identification of immobilized recombinant growth factors.


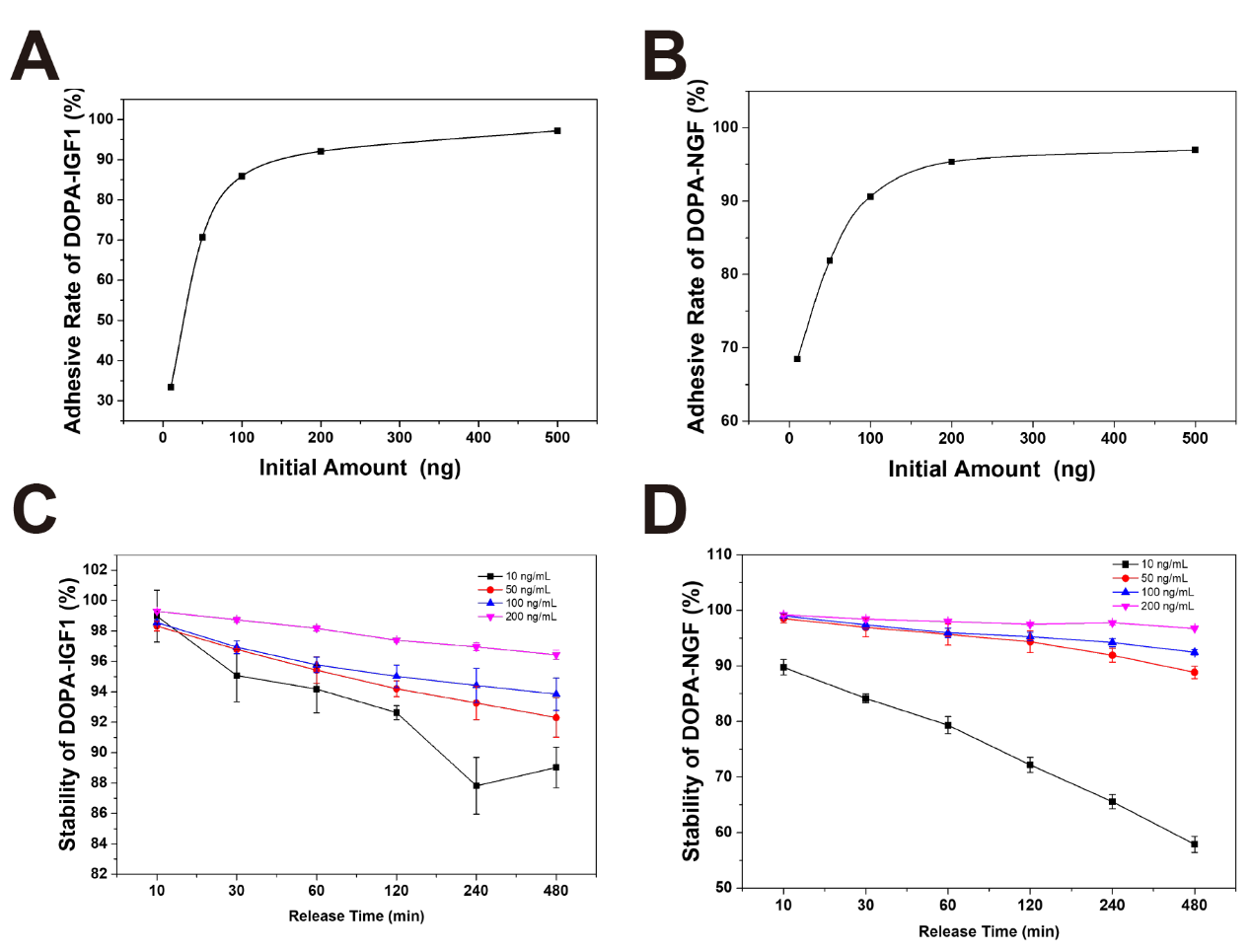


F S2. Adhesion efficiency and stability of immobilized recombinant growth factors. Data are shown as the mean ± SD, n=3.


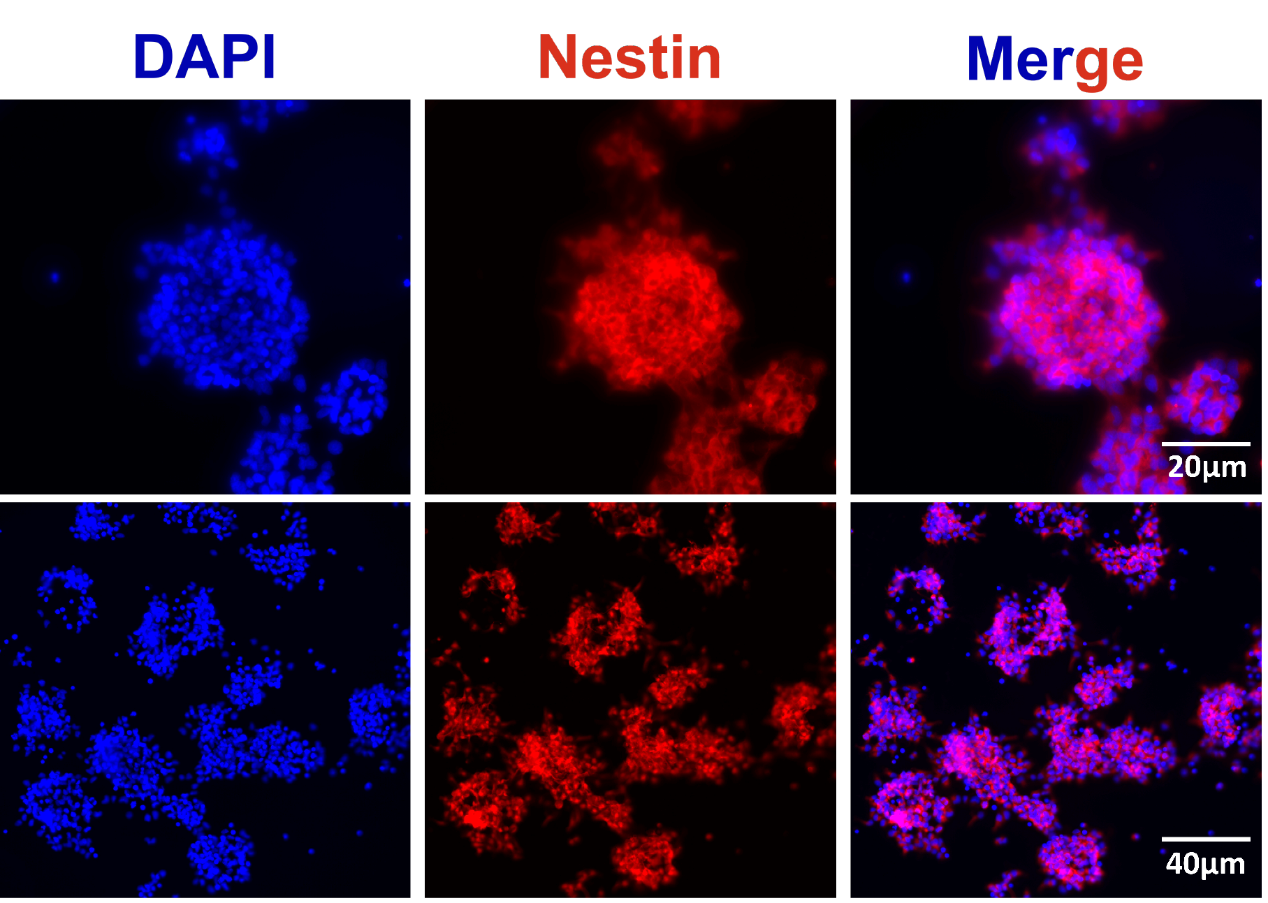


F S3. Immunofluorescence staining of nestin for NSC. Scale bars, 20μm, 40μm.


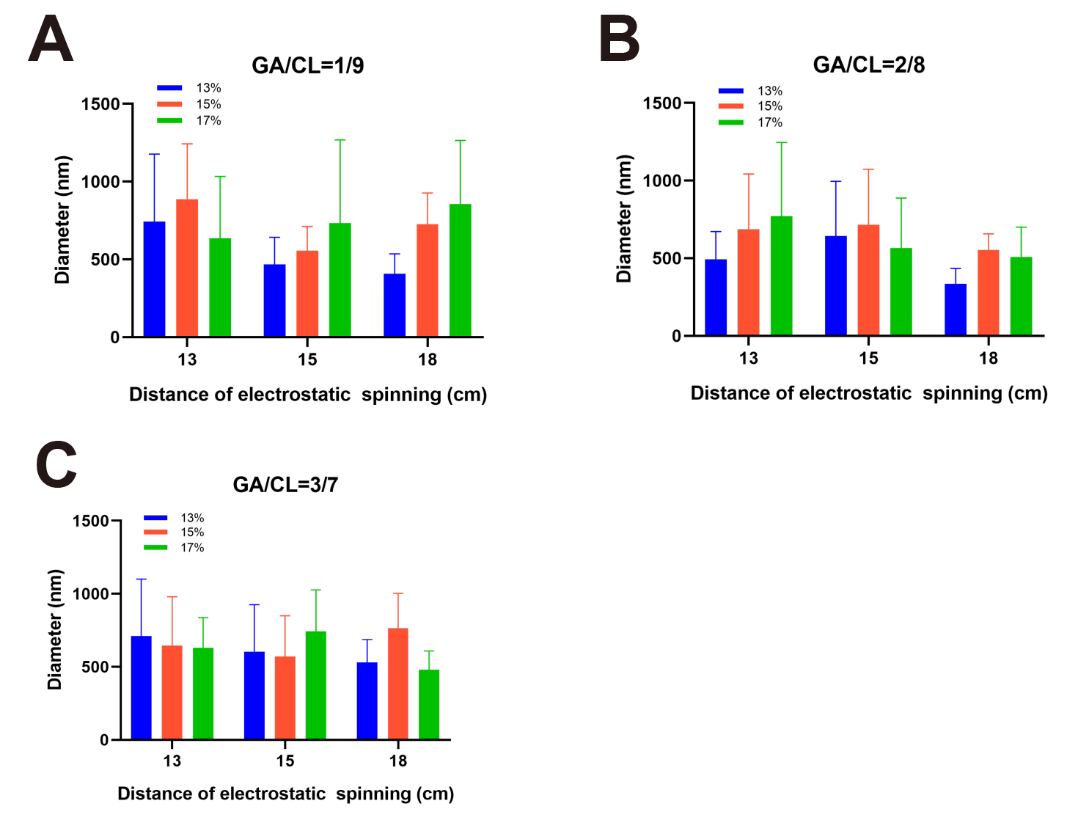


F S4. Statistical Analysis of Fiber Diameter of Scaffolds Prepared Under Different Process Parameters. Data are shown as the mean ± SD, n=100.


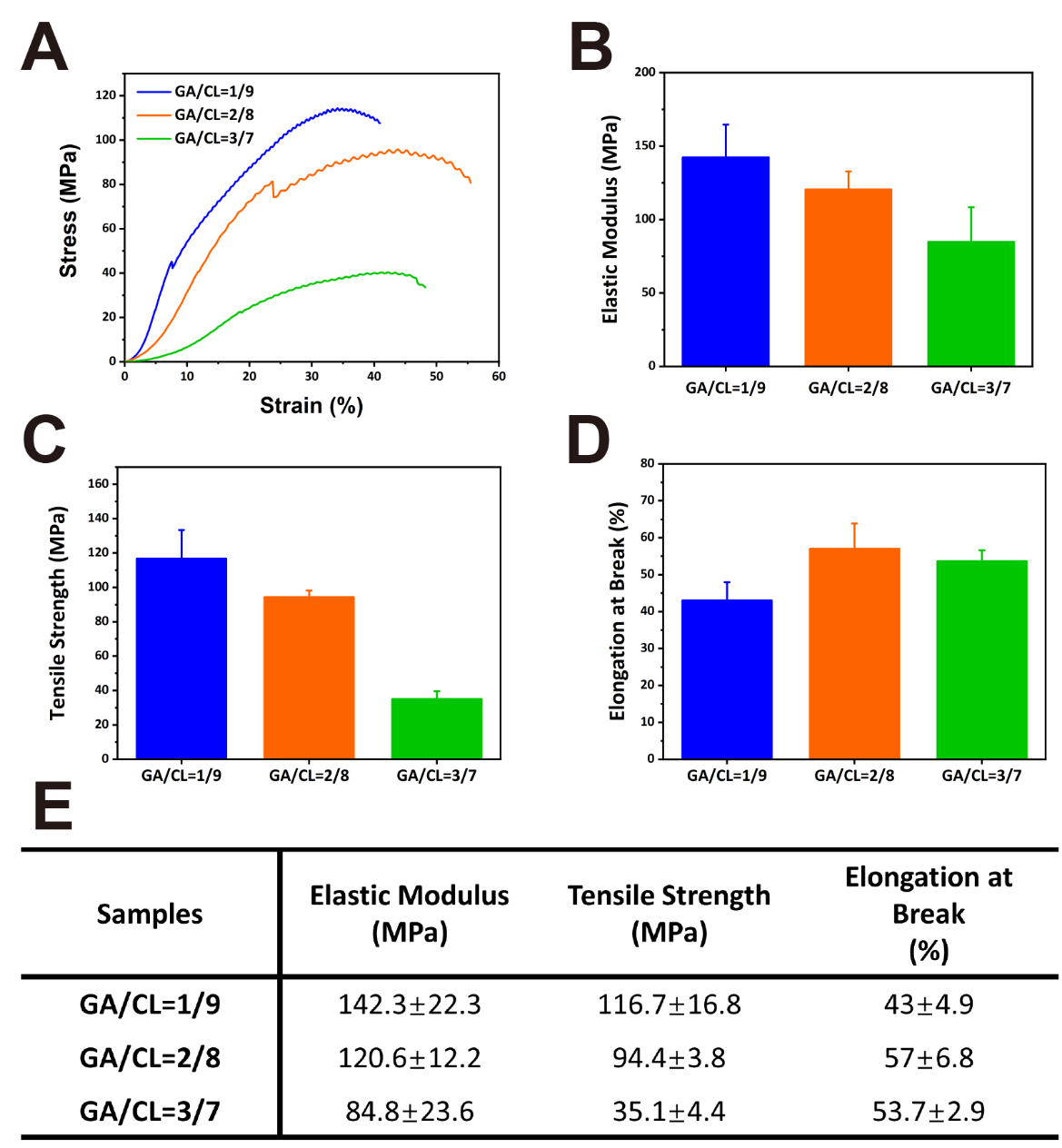


F S5. Mechanical properties testing of fiber scaffolds in different groups. Data are shown as the mean ± SD, n=3.


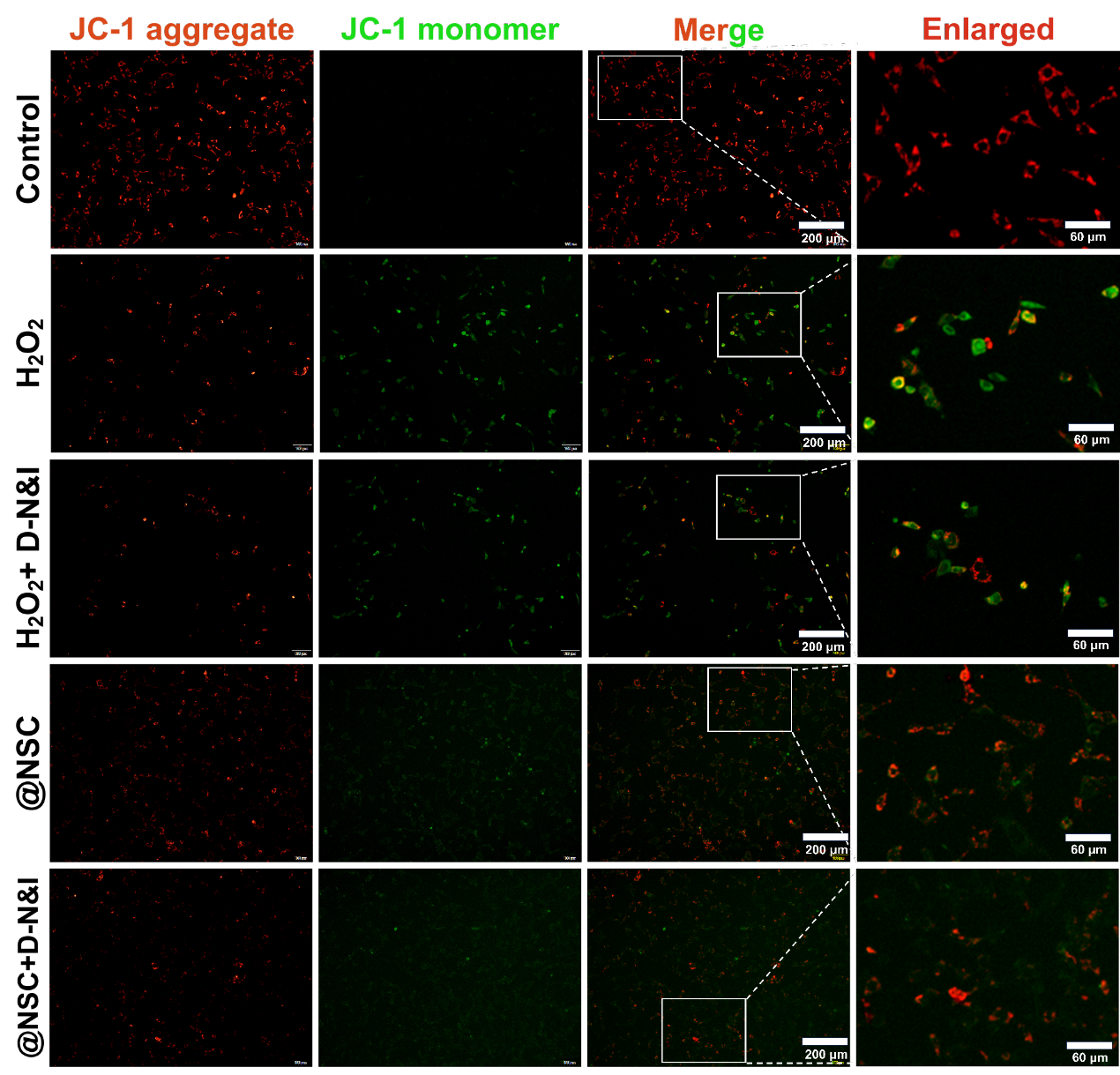


F S6. Mitochondrial membrane potential detection of HT22 co-cultured in the Transwell system under different treatment groups. Scale bars, 200μm, 60μm.


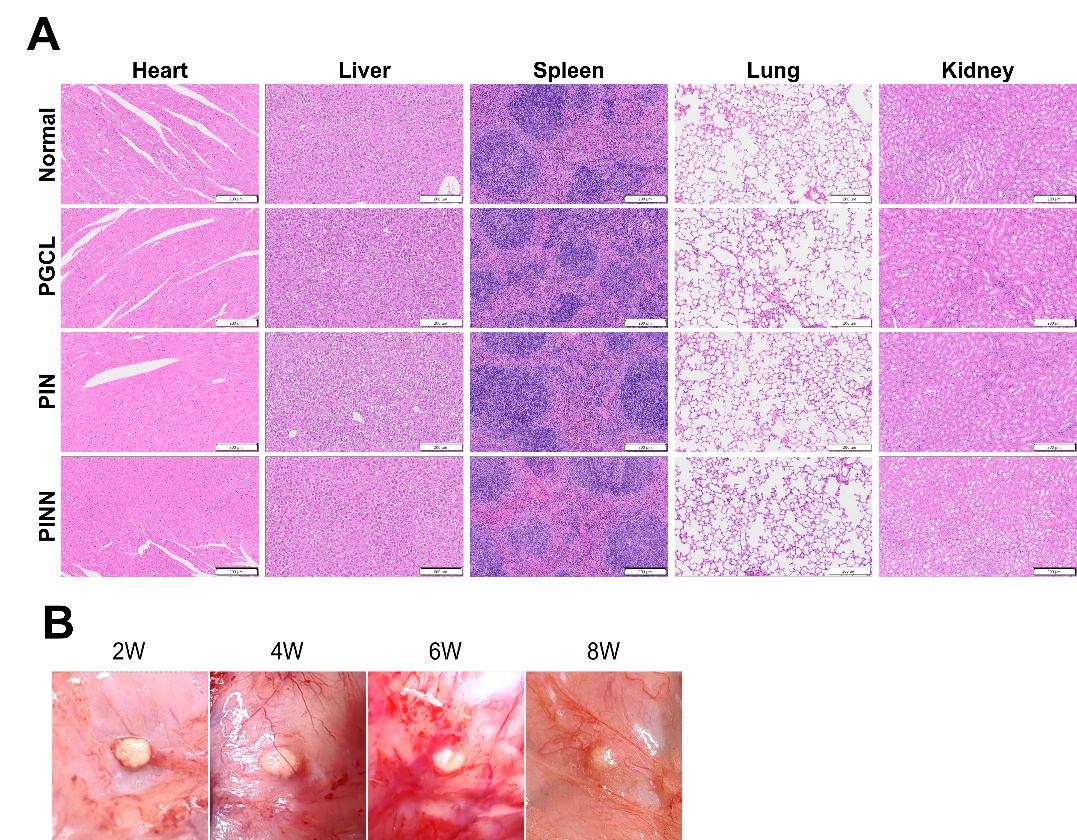


F S7. A The biosafety of the scaffold in vivo experiments. Scale bars, 200μm.

B In vivo degradation behavior of the scaffold.

I


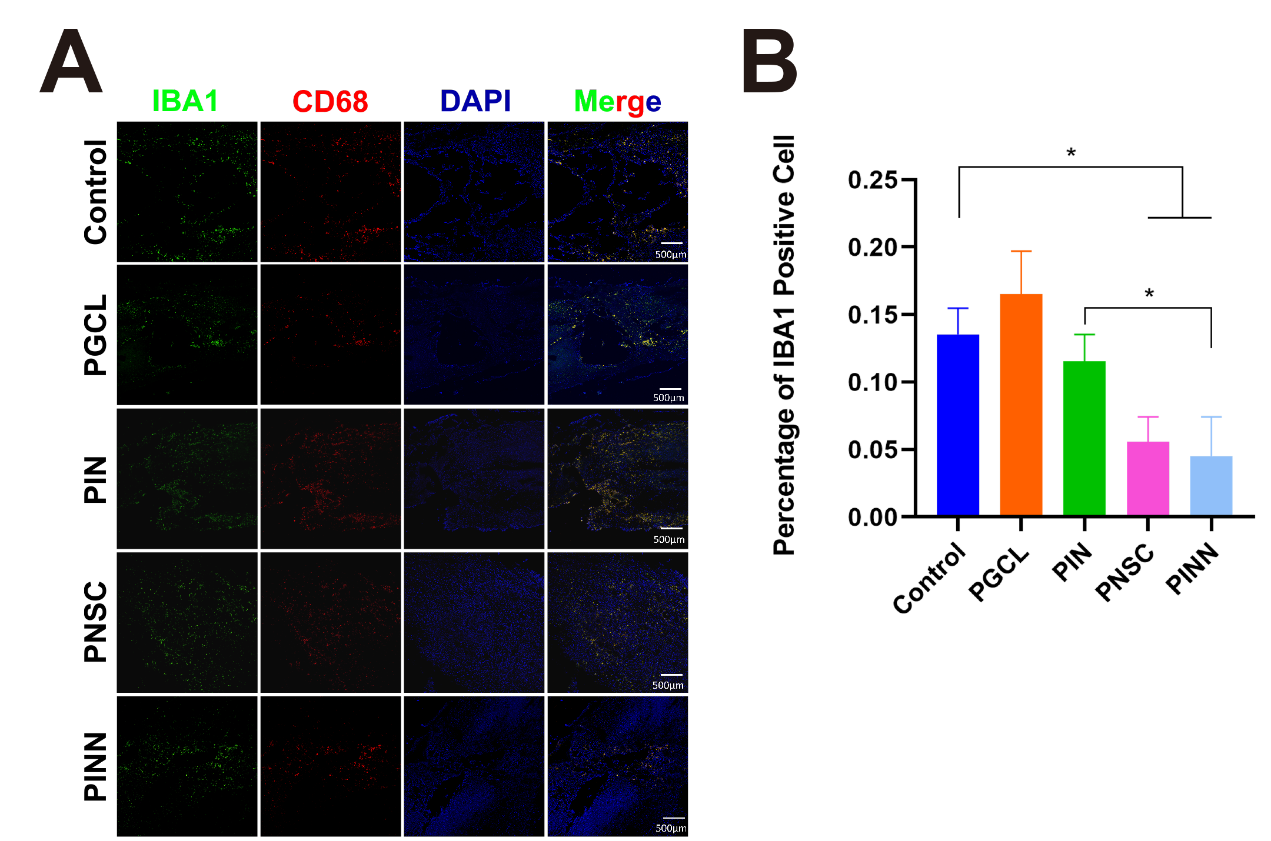


F S8. Immunofluorescence Staining of IBA1 (Green) and CD68 (Red) to Observe and Analyze Inflammatory Response in the Injury Site of Spinal Cord Tissue in Different Groups. Scale bars, 500μm. (Data are shown as the mean ± SD, n=3, *p < 0.05)


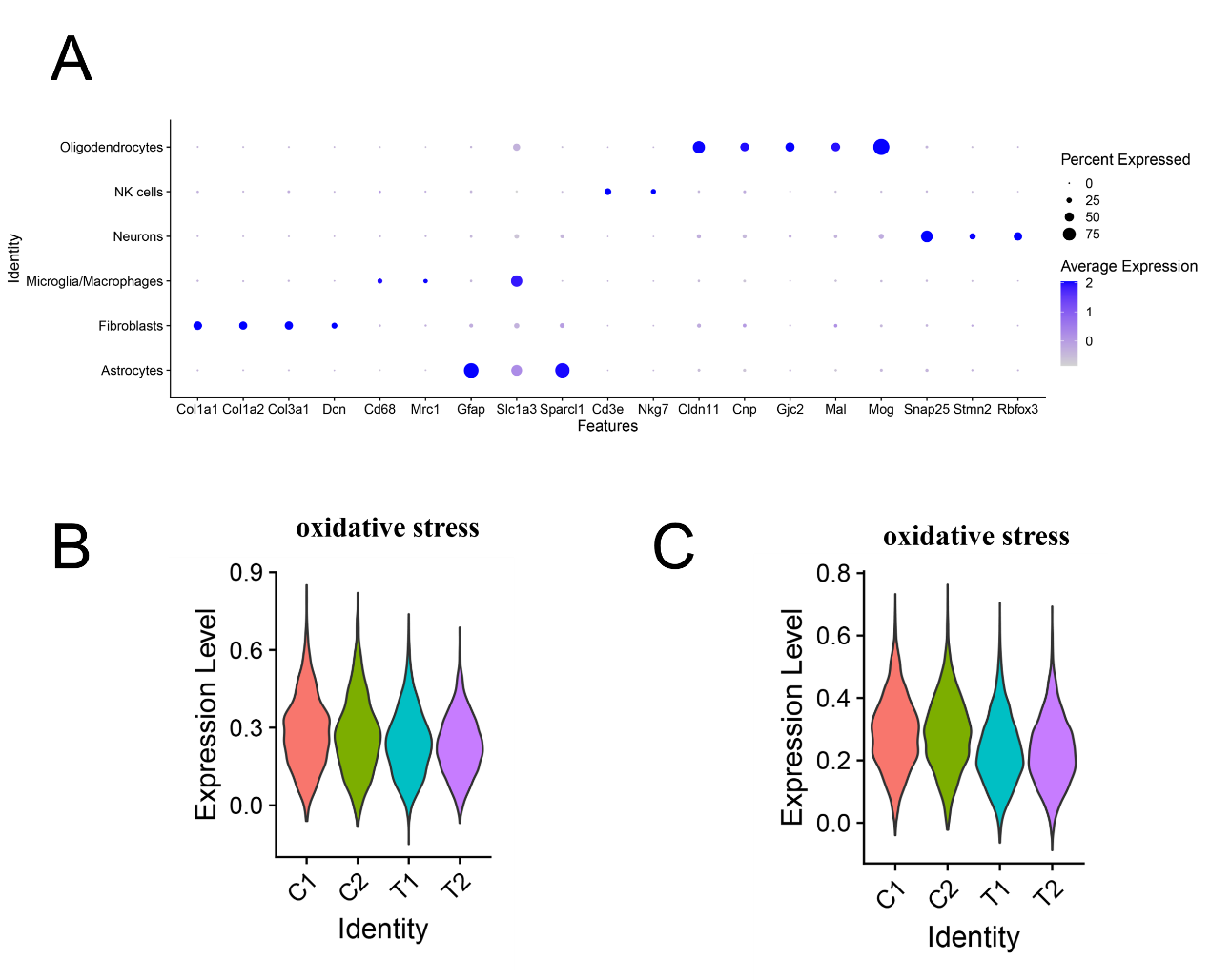


F S9.

(A) Cell type-specific markers for spinal cord tissue annotation.

(B-C) ssGSEA analysis revealed a significant reduction in the activation of oxidative stress-related genes in neurons and oligodendrocytes following treatment.


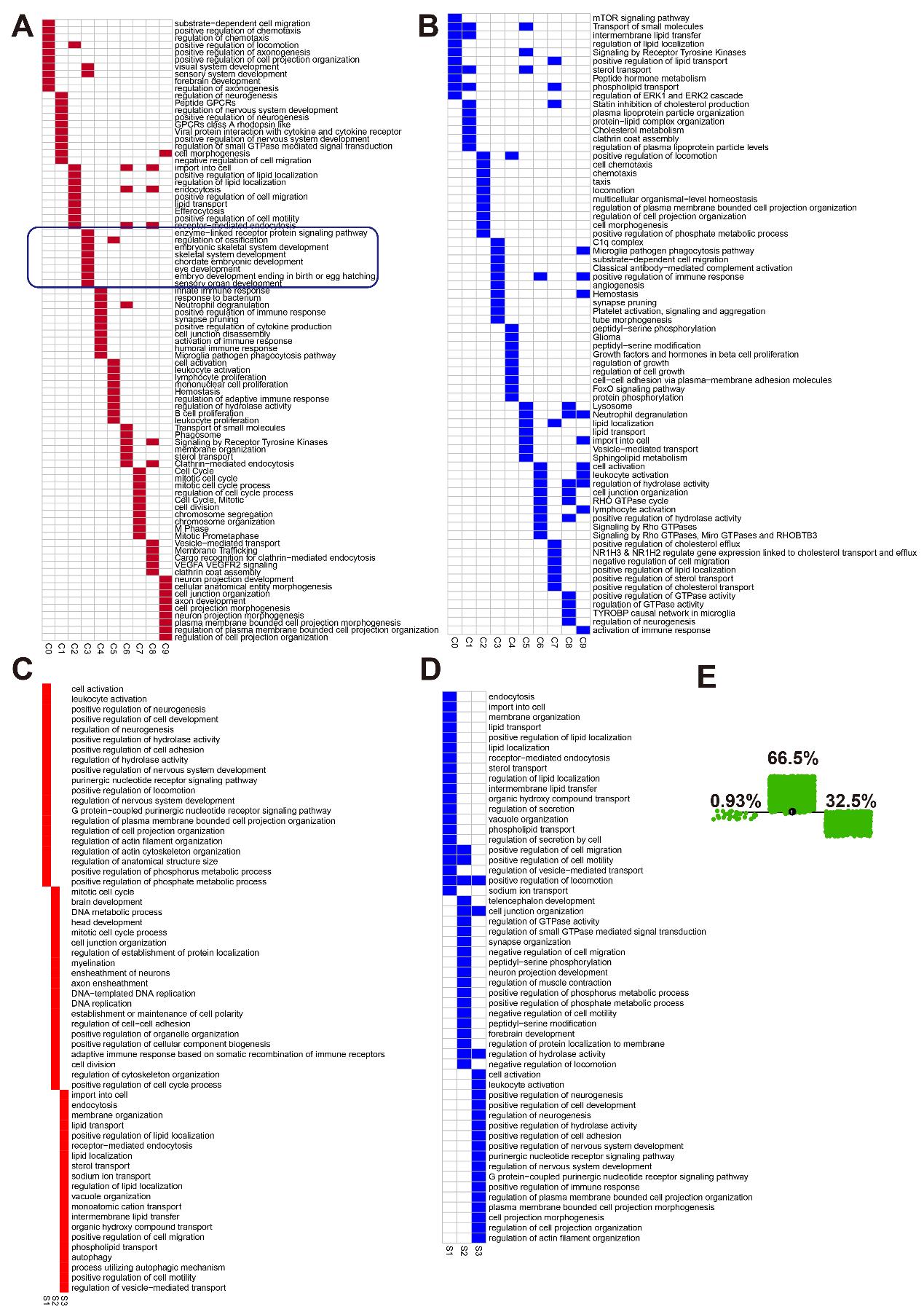


F S10. GO analysis results related to microglia/macrophages.

(A-B) GO analysis revealed the enriched and downregulated biological processes in the differentially expressed genes of microglia/macrophage subtypes.

(C-D) GO analysis identified the enriched and downregulated biological processes in the differentially expressed genes of microglia/macrophages across three developmental states.

(E) The developmental state of the M3 subset of microglia/macrophages.


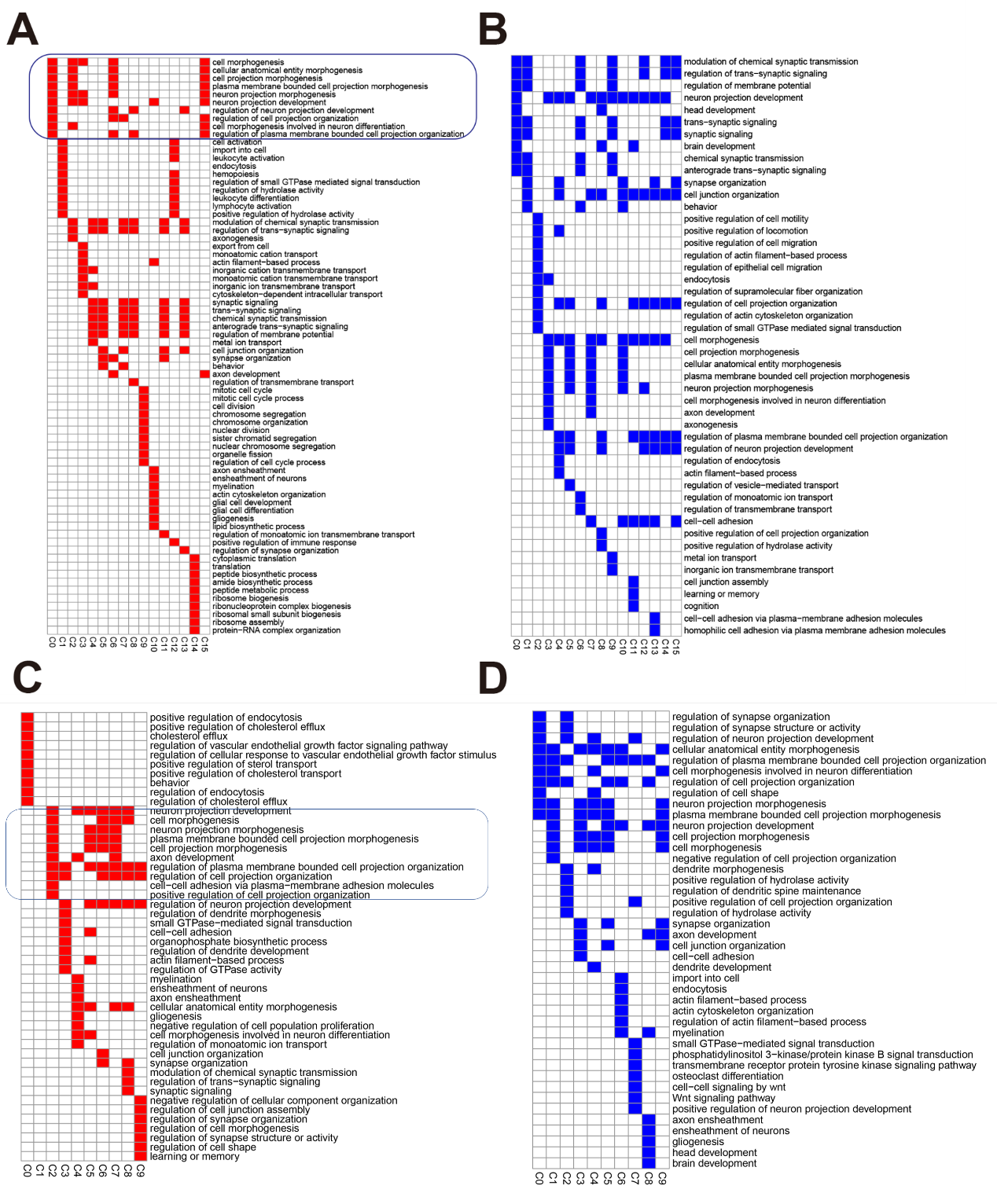


F S11. GO analysis results related to subtypes of neuron and oligodendrocyte.

(A-B) GO analysis revealed the enriched and downregulated biological processes in the differentially expressed genes of neuron subtypes.

(C-D) GO analysis revealed the enriched and downregulated biological processes in the differentially expressed genes of oligodendrocyte subtypes.


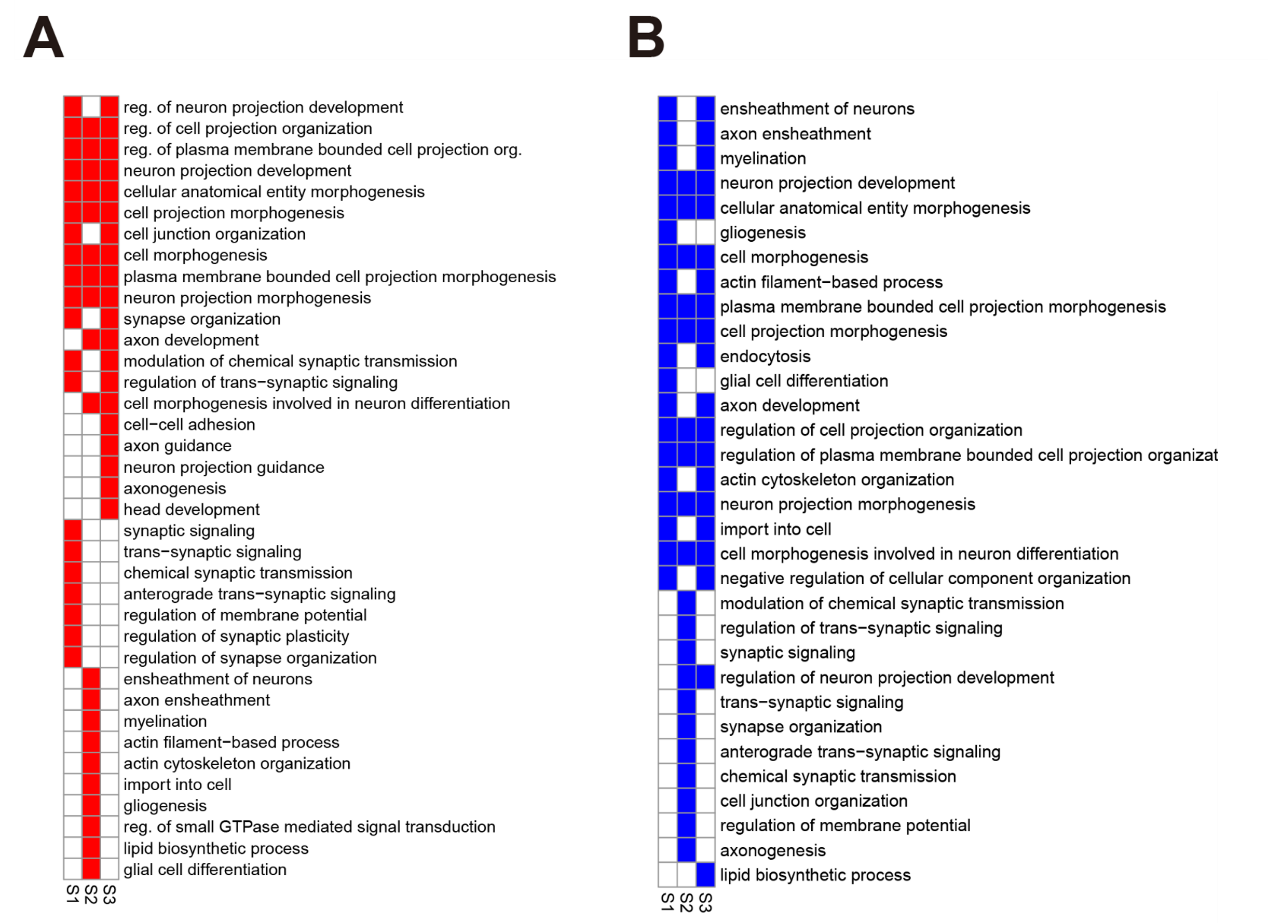


F S12. (A-B) GO analysis identified the enriched and downregulated biological processes in the differentially expressed genes of neuron and oligodendrocyte across three developmental states.


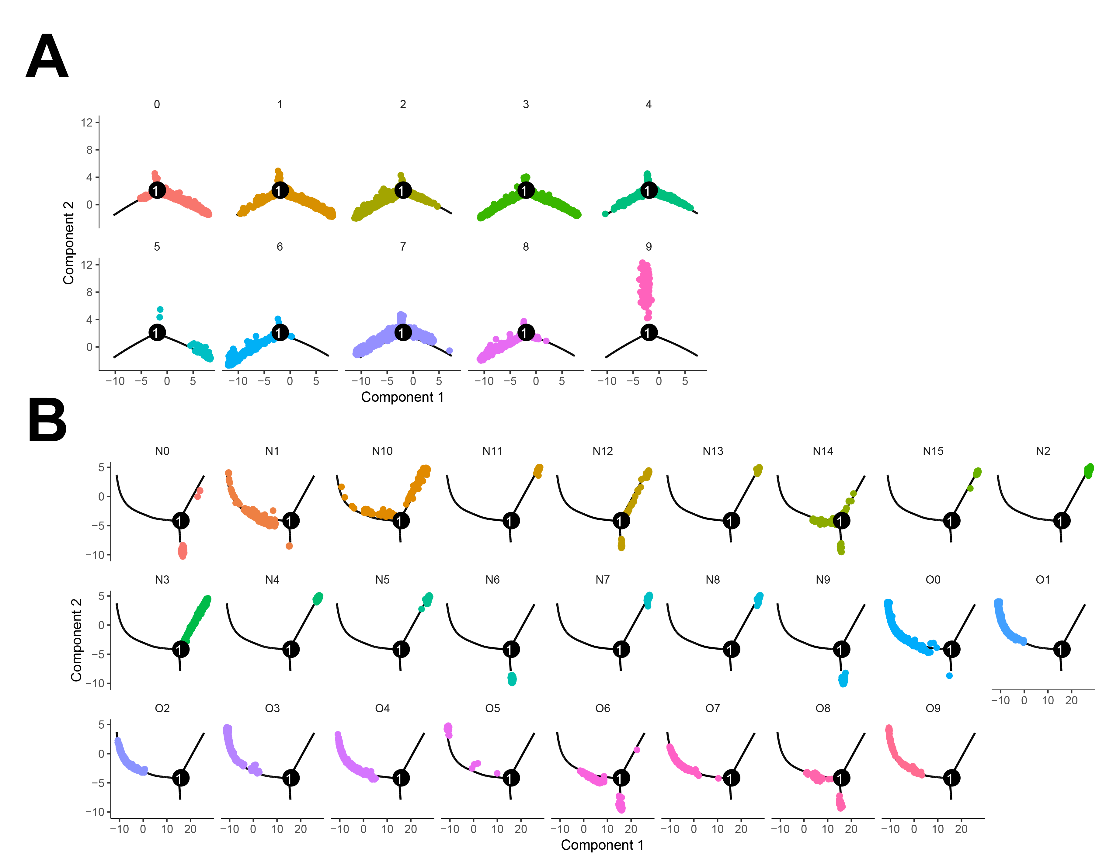


F S13. A The differentiation trajectories of 10 microglia/macrophage subtypes.

B The differentiation trajectories of 26 neurons and oligodendrocytes subtypes.


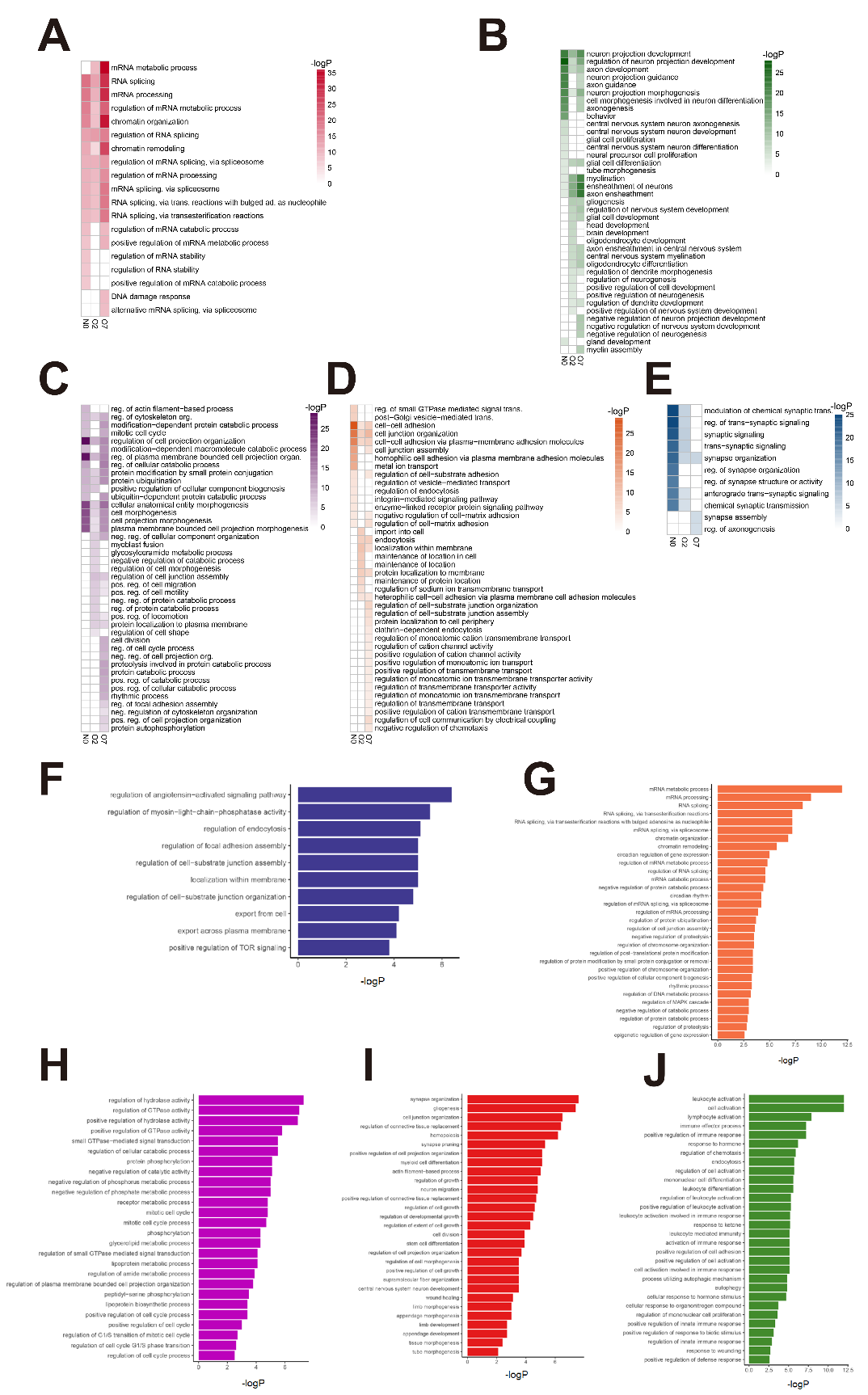


F S14. grn分析揭示损伤区域小胶质细胞/巨噬细胞、神经元、少突胶质细胞特异性亚群M3、N0、O2、O7的生物功能. A,B,C,D,E GO enrichment analysis for regulatory networks of neuron subtype N0, and oligodendrocytes subtypes O2, O7. In the figure, the color red signifies rna binding processes, green denotes nervous system development processes, purple indicates cell morphological changes processes, orange represents signal transduction processes, and blue symbolizes synapse formation. The heatmap values reflect the maximum -log P value for each term across all gene modules within the respective cell subtype. F,G,H,I,J GO enrichment analysis results of functional modules identified by the microglia/macrophage subtype M3 gene regulation network using the Deepwalk algorithm combined with the GMIGAGO algorithm.


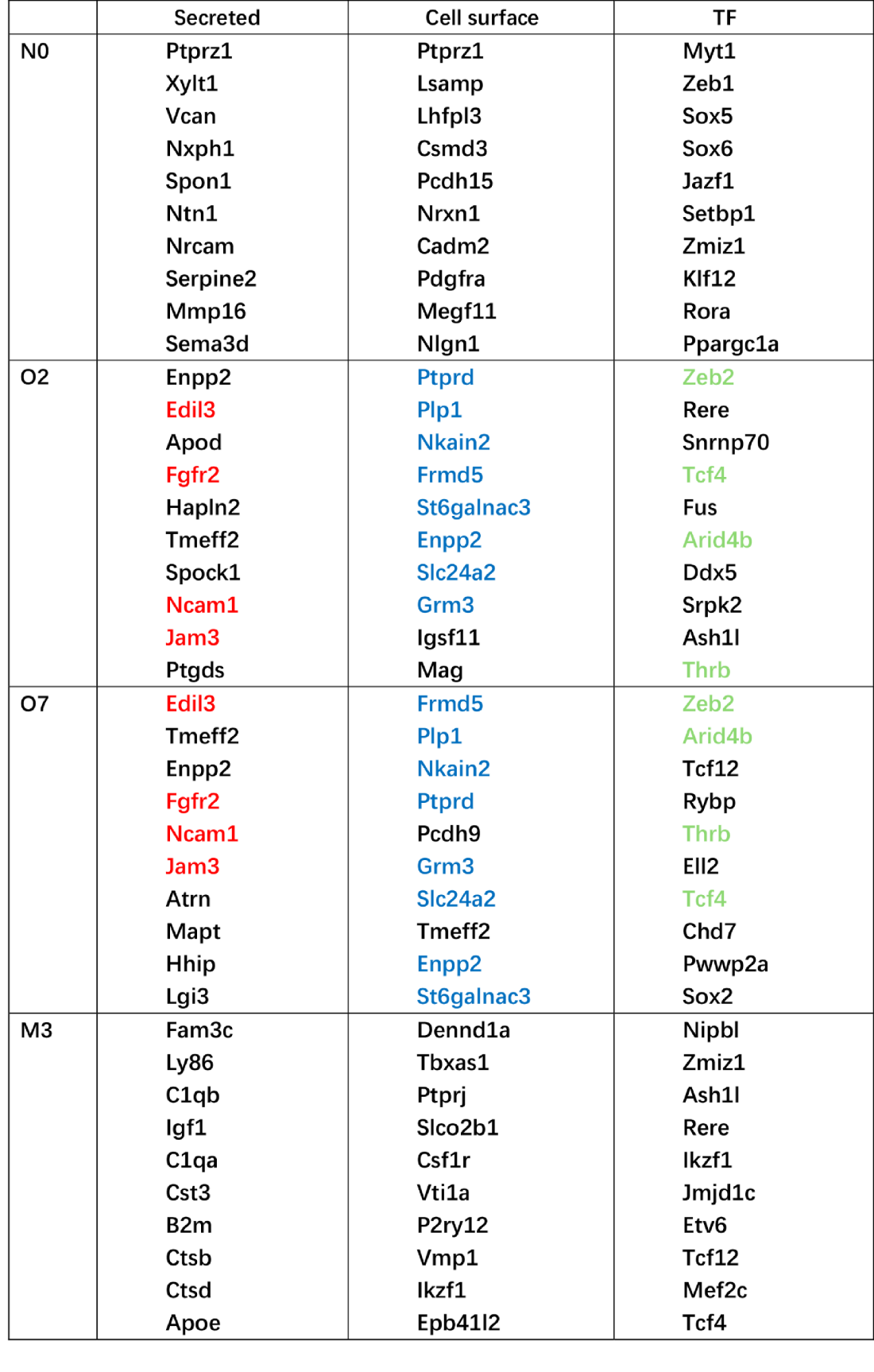


F S15. The expression profiles of the top 10 driving genes in key microglial/macrophage (M3), neuronal (N0), oligodendrocyte (O2), and oligodendrocyte precursor (O7) subtypes, including secretory proteins, transcription factors, and surface membrane proteins.
